# Small Ruminant Production Based on Rangelands to Optimize Animal Nutrition and Health: Building an Interdisciplinary Approach to Evaluate Nutraceutical Plants

**DOI:** 10.3390/ani10101799

**Published:** 2020-10-03

**Authors:** Rafael Arturo Torres-Fajardo, Pedro Geraldo González-Pech, Carlos Alfredo Sandoval-Castro, Juan Felipe de Jesús Torres-Acosta

**Affiliations:** Facultad de Medicina Veterinaria y Zootecnia, Universidad Autónoma de Yucatán, Mérida 97000, Yucatán, Mexico; rafael-arturo-torres@outlook.es (R.A.T.-F.); pedro.gonzalez@correo.uady.mx (P.G.G.-P.); tacosta@correo.uady.mx (J.F.d.J.T.-A.)

**Keywords:** anthelmintic plants, gastrointestinal nematodes, goat, interdisciplinary approach, native vegetation system, nutraceutical, plant-herbivore-parasite interactions, plant secondary compounds, sheep

## Abstract

**Simple Summary:**

The low deciduous forest is an ecosystem covering 8% of México’s surface and is present in 15 of the 32 states of the country. It has been observed that sheep and goats feeding in this high biodiverse biome can consume up to 80 plant species. In addition, the low deciduous forest biomass is frequently used to feed small ruminants in cut and carry systems. However, there is not enough information to provide guidelines for its sustainable use by ruminant livestock. In the present review, we present an interdisciplinary approach aimed at identifying the nutraceutical properties of this native vegetation system, involving disciplines like botany, ecology, agronomy, ethology, ethnoveterinary, nutrition, parasitology and chemistry. Nutraceuticals are defined as livestock feeds combining nutritional value with beneficial effects on animal health and productivity. The identification of nutraceutical properties amongst plant species of the low deciduous forest may contribute to the revalorization of this native vegetation system and provide information enabling the design of sustainable livestock feeding/management systems that benefit the nutrition and health of small ruminants, and ultimately human health through the consumption of animal products produced in this native vegetation system.

**Abstract:**

The plant kingdom can influence the productivity and health of herbivores at different levels. However, demonstrating this process in a scientific manner entails substantial endeavors from different disciplines. In the present review, we will describe the features of a native vegetation system traditionally used by small ruminants and use its particularities to build an interdisciplinary approach to evaluate the nutraceutical properties of plants. Initially, we will establish the context of the low deciduous forest (LDF), considering some botanical and nutritional aspects, as well as the presence of plant secondary compounds (PSC) and gastrointestinal nematodes (GIN). Furthermore, we will focus on coevolutionary aspects that undoubtedly shaped the plants–nutrients–PSC–GIN–herbivore relationship. In addition, the concept of nutraceutical will be discussed to provide clarity and aspects to be considered for their evaluation. Then, ethological, agronomical, nutritional, PSC, parasitological and animal species issues are deepened placing emphasis on methodological approaches. Special focus is given to condensed tannins, as they are the fourth largest group of PSCs and the most studied in livestock sciences. Validation of the nutraceutical properties of plants from native vegetation systems should be seen as a process derived from many scientific disciplines that feed into each other in a cyclic manner.

## 1. Introduction

Projections suggest that by 2030, domestic ruminant numbers in developing countries will exceed those of the entire planet in 2000 [1]. Meeting the booming demand for livestock and livestock products, therefore, requires the development of more efficient, sustainable and alternative feeding systems to support the future forage requirements, which cannot be sustained by single species grass-feeding systems [2]. Rangelands are native vegetation environments with heterogeneous vegetation cover including grasses, creepers, shrubs and trees. These ecosystems have served as a source of food for livestock for centuries [3,4]. In many parts of the world, these systems provide the main forage resource for traditional livestock [5,6] or represent the only commodity available to very poor communities [7], affecting the livelihoods of millions of people.

However, commonly most people consider rangelands as a “waste-land” because its conditions are unfit for many human agricultural activities [8,9]. In some parts of the world, wealthy farmers destroy rangelands to introduce grass plantations, which are considered as the “way forward” for the modern production of milk and meat from ruminants [10,11,12]. Thus, most common people look at the native vegetation as a useless resource, including plants commonly classified as “weeds”, which are used by the poor and the backward. However, those ideas must be countered from the standpoint of solid scientific knowledge. Rangelands are valuable in their own right for the large number of organisms they include as well as their complex interactions, which we are only beginning to discover and understand. Some rangeland ecosystems, such as the low deciduous forest (LDF) include plants that are important for carbon sequestration, building, ceremonial activities, medicinal purposes, crafting materials, fuel and production of nectar and pollen for beekeeping [13,14,15]. Furthermore, recent studies exploring the nutritional value of the plant species in the LDF also cast light on its potential value for ruminant livestock. Thus, efforts to incorporate strategies aimed at maintaining long-term productivity and convert native vegetation biomass into valuable food products for humans continues to be a focus of animal scientists [4,12].

In this context, the LDF could represent a viable scenario for research purposes. The LDF vegetation in México represents an important source of nutrients for most small ruminant flocks, and in some cases represents the only source of feed [13,15]. Over 200 LDF plant species have been reported to possess feeding potential for domestic ruminants [16]. However, coping with such a level of heterogeneity may not represent an easy task for sheep and goats, given that, on a daily basis, they are confronted with the challenge of establishing a feeding strategy through the selection of various plants containing different arrays of nutrients and plant secondary compounds (PSC) in different concentrations in large and constantly changing permutations [1,17,18]. Nevertheless, smallholders have taken advantage of the ability of small ruminants to thrive in these ecosystems, harvesting (grazing/browsing) its biomass to obtain their nutrient requirements. Sheep and goats have been in a coevolutionary “arms race” against plant defense mechanisms for millennia. Hence, they need to develop adaptive mechanisms [19,20,21] not only aimed at coping with diverse levels and concentrations of nutrients and PSC present in plants, but also at obtaining certain benefits after their consumption.

Indeed, a large body of research has focused on the capacity that some PSC have to alleviate or prevent animal diseases. Considering that conventionally, the gastrointestinal nematodes (GIN) infections are considered as one of the most significant constraints for small ruminants feeding on natural scenarios [22,23,24], efforts have been directed to assess, under in vitro and in vivo approaches, the anthelmintic (AH) effect of some PSC against the different life stages of GIN. The bulk of the research has been directed at identifying the AH activity of the condensed tannins (CT) [25,26,27,28,29,30,31], while some PSC, like saponins, flavonoids, terpenoids, alkaloids and sesquiterpene lactones, have also been studied more recently [32,33,34]. In the meantime, the growing evidence on the relationships between herbivores, plants, PSC and GIN served as the springboard for the introduction to the novel “*nutraceutical*” concept in the veterinary sciences. According to Hoste et al. [35], a nutraceutical can be defined as a livestock feed which combines nutritional value with beneficial effects on animal health. The latter situation bolsters the idea that native plants harvested by sheep and goats could be considered as nutraceuticals, since they inherently contain different arrangements of nutrients and PSC. To date, however, there are few approaches to identifying plants with nutraceutical properties despite the large biodiversity of rangelands, which may be partly due to the lack of specific guidelines [35]. Considering the latter, the identification of the nutraceutical value of plant species would be a key factor to consider when developing feeding strategies and management schemes that promotes the sustainable use and preservation of rangelands.

In the present review, we will summarize key points of the specific context present in the LDF of México. Further, we will explore the concept of nutraceutical based on the context of this vegetation system. Finally, we will focus on an interdisciplinary approach aimed at identifying plants with nutraceutical properties. Validation of the nutraceutical properties of rangelands could contribute to the design of more sustainable livestock feeding/management programs that optimize the nutrition and health of sheep and goats. An additional outcome would be the revalorization of the LDF plant resources by farmers, general population and political stakeholders alike, who undoubtedly must contribute to protect or rationalize the use of this ecosystems.

## 2. Unravelling the Heterogeneous Context of the Low Deciduous Forest

The LDF is the most widely distributed tropical vegetation of México, and even the largest of this type in Latin América [36]. It has been estimated that approximately 60% of the plant species of this ecosystem are exclusively from México [37], hence, its ecologic and conservation value has been highlighted [38,39,40]. The LDF covers approximately 8% of México’s surface area (~157,000 km^2^), being present in some portions of the states of Baja California, Campeche, Chiapas, Colima, Estado de México, Guerrero, Michoacán, Morelos, Nayarit, Oaxaca, Puebla, Sonora, Tamaulipas, Veracruz and Yucatán [41]. In Yucatán, the LDF is the most widely distributed plant community and, along with the medium semi-deciduous forest, typifies the physiognomy of the state’s landscape, covering an extension of ~20,000 km^2^ [42] which are distributed in the center, west and extend in a non-uniform strip from the northeastern portion of that state, reaching the neighboring Campeche state. The LDF thrive in sub-humid, semi-arid and warm climates, with a median annual temperature above 20 °C and an average annual rainfall between 700 and 1200 mm [36,40], and is characterized, initially, for its arboreous component not exceeding 15 m in height, as well for the high percentage (>70%) of plant species with deciduous leaf’s falling during the dry season [38]. Thus, the interannual rainfall is the primary determinant of foliage productivity [4,43].

### 2.1. Botanical and Nutritional Components of the Low Deciduous Forest

The LDF include plant species with nutritional features that are employed by small ruminant farmers (as grazing/browsing paddocks or in cut and carry systems). The LDF serves as a source of foliage during the dry and the rainy season, with plants of medium to high nutritional value, in various life forms and heights. Several species have been reported to contain PSC which could provide an added health value, leading to an enhanced animal productivity [14]. According to some estimates, the number of plant species in the Yucatán peninsula reaches about 2200 [44]. In a 10-year survey, it was determined that Mayan communities use 196 plant species as forage (for cattle, sheep, goats, pigs and poultry), amongst which 139 were herbaceous, 17 were shrubs, 35 were trees and 2 were palms [3]. Similarly, the LDF vegetation in the center-north region of Yucatán is comprised of 123 plant species belonging to 41 plant families [41]. Despite this plethora of botanic families, the Fabaceae is the best represented [36]. The presence of other families, such as Convolvulaceae, Malvaceae, Asteraceae, Polygonaceae, Rubiaceae, Boraginaceae, Verbenaceae, Euphorbiaceae, Acanthaceae, Cactaceae, Burseraceae, Compositae, Malpighiaceae and Anacardiaceae, was also reported [36,42].

A common feature of the Fabaceae (also known as Leguminosae) family, is their high content of crude protein (CP) and PSC [42], although plants from other families could also contain high macronutrient or PSC contents. For instance, *Gymnopodium floribundum* and *Neomillspaughia emarginata* (Polygonaceae) present 37.6 and 37.5% CT, respectively, and conversely, *Viguiera dentata* (Asteraceae) and *Ipomoea crinicalyx* (Convolvulaceae) present 29.6 and 23.7% CP, respectively. Previous studies detailed the macronutrient and PSC characterization of plant fodder [45,46] and pods [47] consumed by small ruminants in the LDF. On the other hand, vegetation from the LDF, normally present low energy content, which is mainly provided by grass species. This situation is worsened because the latter have a marked seasonal availability, with most dying during the dry season [48]. Considering these particular features, we can state that LDF represents a source of high dietary CP with a limiting energetic value. Hence, energy-rich dietary supplementation has been proposed to optimize its use by sheep [49] and goats [50].

### 2.2. Plant Secondary Compounds in the Low Deciduous Forest

According to Estell et al. [1], the literature is replete with studies on the role of PSC in herbivores for both domestic livestock and wildlife on every continent. Although a thorough analysis of the PSC nature is beyond the scope of the present review, we want to point out some key aspects that deserve to be addressed in order to build a more comprehensive panorama of these compounds, the context in which they are present and the animal species that eventually incorporate them into their diets.

Plants, as sessile organisms, have no chance of escaping attacks from other organisms, so they must employ other strategies to defend themselves [17], including a number of chemical components known as “natural compounds”, “phytochemicals”, “plant secondary compounds” or “specialized metabolites”, the last two terms being the most accepted within the veterinary and ecological sciences, respectively [31,51]. The PSC are synthetized in the whole spectrum of the plant kingdom and their presence and concentration in a given plant is influenced by genetics, phenology, and a variety of biotic and abiotic factors [21]. Estimates suggest there are up to 200,000 PSC produced by different plants in response to particular challenges [52]. However, some authors argue that this number is probably a gross underestimation given the low number of plant species investigated to date and the low concentrations of some specialized PSC, which in turns affects the effectiveness of their detection [51].

From an ecological perspective, PSC represent adaptations to specific situations that facilitate interactions with the biotic and abiotic environment, including the essential role of chemical defenses against pathogens and herbivory [17,51,52,53]. It worth mentioning that the term *herbivore* comprises a wide range of species, including insects, birds, reptiles, non-ruminant and ruminant mammalians. Indeed, plants and insects have coexisted for at least 350 million years [54], while the current ruminant families entered in the chronology of ecosystems only 18–23 million years ago [55], which likely modifies the dynamics and PSC production in landscapes by incorporating a different type of pressure than that of insects (ruminant liveweights ranges from about 2 to more than 800 kg) [54]. Therefore, the long-term coevolution between herbivores and PSC necessarily entails the triggering of adaptive responses, which correspond mainly to behavioral and physiological mechanisms that involve both pre-ingestive and post-ingestive processes. Those same mechanisms were used to their full extent when domestic ruminants were introduced to the American continent or to islands such as Australia in more recent years, forcing animals to implement different adaptation mechanisms that have only evolved within the last 500 years [6]. We encourage readers to consult reviews in this subject in order to gain a complete perspective of the mechanisms by which ruminants cope with PSC [18,21,56]. Under this context, we emphasize and support the way of thinking of Mueller-Harvey et al. [31] and Villalba et al. [57] when they argued that PSC are components with multiple and interrelated functions looking to improve resilience, provide plasticity and support the development and interaction of plants with their environment.

### 2.3. The Unavoidable Occurrence of Gastrointestinal Nematodes in the Low Deciduous Forest

Regardless of place and climatic conditions, gastrointestinal nematodes (GIN) are a common component of grazing vegetation systems used by sheep and goats worldwide. When domestic ruminants were introduced to the LDF, they also brought their own internal and external parasites. Those parasites also had to become adapted to the new conditions to which they were exposed, particularly the free-living phases that must survive outside the host [6]. The level of success in the survival of parasites in different environmental conditions is the main cause of differences in the array of parasite species present in each region of the world. Thus, although the presence of GIN is common for all the grazing ruminant production systems, the species present in each region vary considerably [58,59]. Under the hot sub-humid conditions of the LDF in México, small ruminants are mainly infected with *Haemonchus contortus* in the abomasum, *Trichostrongylus colubriformis* in the small intestine and *Oesophagostomum columbianum* in the large intestine, with the eventual presence of *Strongyloides* spp., *Trichuris* spp. and *Cooperia* spp. [60]. The mismanagement of grazing schemes may lead to a vicious cycle of land over-exploitation, in which plants’ equilibrium is altered by ruminant herbivory and substantial quantities of infective stages accumulate within the pastures. Ultimately, the accumulation of unsustainable GIN burdens inside the host triggers a condition which is recognized as one of the main constraints for small ruminant production throughout the world [61,62].

There are some noteworthy key epidemiological processes related to the development and viability of GIN that take place within the pastures. Initially, the ruminant host sheds GIN eggs via fecal excretion, and after that, eggs hatch in the feces, releasing larvae, which undergo a moulting process from larvae 1 to larvae 2, to finally become a sheathed larva (on pasture) able to re-infect hosts (larvae 3 or L_3_). This process is largely influenced by some environmental factors, including temperature, humidity and solar radiation [63,64]. Additionally, it is important to consider that both the nutritional resources and the infective L_3_ are unevenly distributed in both space and time [65,66]. Thus, complex decisions must be taken by ruminants in order to obtain enough nutrients while avoiding higher intake of L_3_ [67].

It is assumed that L_3_ are associated with the grass species, but not with shrubs or higher stratum plant species. Experiments conducted in grass monocultures showed that 80% of L_3_ were located in the first 5 cm above the ground [68], although others found a homogeneous distribution of L_3_ in different pasture strata [69]. A recent study performed in Brazil reported no differences in L_3_ content between two grazing systems (grass vs. grass + legumes) using the pasture larval count technique (PLCT). However, that study used a short legume that grew mixed within the grass [70]. In heterogeneous vegetation systems such as the LDF, the dynamics of L_3_ migration may represent a distinct pattern. Recent tracer studies suggested that the consumption of high strata fodder (>50 cm) was negatively associated with GIN infection of tracer kids [71]. Sheep and goat production in LDF is usually developed without proper plant management other than occasional pruning. Therefore, tracer studies are preferred over the PLTC due to the complexity in the architecture of plants in the LDF, where animals harvest leaves of different plant species at different heights of strata.

Concomitant with the relationship between herbivores and PSC, a co-evolution process of 10–20 million years between herbivores and GIN occurred [72]. Thus, from an evolutionary perspective, both host and GIN shaped a long-term relationship where a large proportion of animals within a flock can endure moderate loads of GIN without compromise to their health or productive status. As a consequence, only a few animals have unsustainable GIN burdens which cannot be tolerated. The latter has been referred as “*aggregation*” and represents a general law of parasite ecology, where the majority of hosts are infected with few or no parasites, whereas a small proportion of hosts are infected with many parasites [73,74]. For the southeast of México, Torres Acosta et al. [75,76] showed that 75–80% of animals in flocks had low GIN burdens and are able to maintain their productive performance without apparent negative effects. Some of those animals with low GIN infections are capable to limit their infections through their immune system and are known to be “*resistant*” to infections [77,78]. Meanwhile, amongst those animals with GIN infections there are several ones capable of enduring the negative impact of their natural GIN infection, while keeping their ability to grow and reproduce, and these are known as “*resilient*” [77,78]. Considering these concepts, it is important to emphasize the notion that GIN infections in small ruminants on production systems based on grazing/browsing natural vegetation are unavoidable, but also biologically affordable.

### 2.4. The Unraveled Context of the Low Deciduous Forest

It is difficult to generalize any concept or paradigm in a context where different levels and concentrations of plants, nutrients, PSC and GIN vary over space and time, and where the only constant is change. Nonetheless, evolutionary trends shaped many interactions between the aforementioned components. The LDF dynamics show a multifactorial mechanism in which plants contain nutrients and defense strategies allowing them to interact with their environment, and these chemical strategies also enable plants to survive to its environments and control herbivory at some level. On the other hand, LDF also bring benefits after their consumption, which allows herbivores to coexist with certain GIN populations, and the latter are returned to the environment through fecal excretion. A long-term consequence of this adaptive process could be the notion that LDF have up to 260 plant species with feeding potential for ruminants [16], amongst which roughly 80 are been consumed by sheep and goats, as confirmed through observational studies [79,80,81]. This scenario would increase its complexity if the farmers practices in terms of using/managing their LDF grazing/browsing areas is to be considered.

## 3. Building an Interdisciplinary Approach to Identify Plants with Nutraceutical Potential in Heterogenous Feeding Scenarios

In this section, we will show an approach to identify and evaluate the nutraceutical properties of plants considering the inherent characteristics of rangelands. Such methodology requires linkage between various disciplines, like botany, ecology, agronomy, ethology, ethnoveterinary, nutrition, parasitology and chemistry, amongst others, as proposed by Hoste et al. [35]. First, we will show the ambiguity in the formal definition of a nutraceutical resource and establish our interpretation. After that, we will address a systematic approach to identify potential nutraceutical plants, considering criteria from the abovementioned disciplines. Last, we will focus on some particularities related to the small ruminant species under study.

### 3.1. The “Nutraceutical” Debate

Introduction of the term “*nutraceutical*” occurred some three decades ago, when the chairman of the foundation for innovation in medicine from New Jersey, USA, coined the word by joining the terms “nutrition” and “pharmaceutical” [82,83]. Since that moment, there was a monumental upsurge in interest in and the popularity of these products in different scientific disciplines, amongst which livestock production and veterinary science were not the exception. At first, nutraceuticals were used more commonly and extensively in humans and non-ruminant livestock than in ruminants [84]. Nowadays, the nutraceutical industry totals more than 250 billion per year (USD), and their use in animal health and diseases is more popular than in humans due to their relatively low cost and safety [85]. Within the livestock sciences, the focus has been on employing nutraceutical materials as tools to reduce the use of antimicrobials, given the increasingly limited therapeutic options to treat sick animals [86]. However, based on the literature review conducted, we noticed the lack of an unified conceptualization about nutraceuticals, which could be partly due to the ample classification systems and/or the lack of regulatory laws/organizations to oversee these products [87], especially when comparing the concepts of nutraceutical between human and veterinary sciences. To make the picture more complex, this never-ending debate include terms like “*chemical additive*”, “*cosmeceutical*”, “*functional food*”, *“phytotherapeutic”* or “*therapeutic food*” [88]. Definitions of these terms are presented in Table 1, and the most common definitions regarding the term nutraceutical are presented in Table 2.

Considering the latter, it is clear that definitions differ from country to country and from author to author, although it seems that nutraceuticals fall somewhere between feed, nutrients and drugs. Maybe a clear borderline would be to consider that a nutraceutical material is not a drug, hence it does not need to comply with all the legal procedures necessary for drug registration in any of the government instances worldwide. However, it is very plausible that the lack of regulation represents one of the main limitations for their implementation, which was widely addressed recently by Gupta et al. [85], who pointed out the regulatory aspects of nutraceuticals in different continents and countries, including North America, the European Union, India, China, Australia, New Zealand, Turkey, the Philippines and South Africa. We encouraged readers to look at this comprehensive text to deepen their perspective on this subject.

Due to its complexity and how wide and certainly ambiguous the classification of a resource as a nutraceutical can be, the present review will be based on two aspects:

- In order to build the approach under a heterogeneous feeding scenario, we will consider the definition by Hoste et al. [35]: “a livestock feed which combines nutritional value with beneficial effects on animal health could be considered as nutraceutical”;
- The existing literature addresses several benefits of nutraceuticals [83,84,85,86,87]. In this review, we took as a basis the threat that GIN represents for small ruminants in grazing scenarios and focused on the AH properties of some LDF plant species, placing emphasis on CT, which are the most studied PSCs in relation to small ruminant nutrition and health. Nevertheless, the AH properties of other classes of PSC will be briefly discussed.

### 3.2. The Ethological and Botanical Approach

To be consistent with Hoste et al. [35], the very first requisite is to ensure that a given plant is indeed freely consumed by animals. Any feed resource that is not eaten cannot be considered as nutraceutical because a nutraceutical is, first and foremost, a feed destined to provide macronutrients, vitamins, minerals and PSC to livestock. Fortunately, there are at least 80 identified plant species selected by small ruminants in the LDF, and thus, are potential candidates for nutraceutical use. To obtain this information, we performed a methodological tool named “continuous bite monitoring through direct observation (DOM)” [93,94]. To the best of our knowledge, this technique is the first choice to quantify the feeding behavior of domestic herbivores under heterogeneous environments. In addition, it is non-invasive and considers the animal’s point of view on resource availability and usage [95,96]. The DOM is the result of gathering many tools such as bite counting [97,98,99], hand-plucking [100,101], construction of a “bite coding grid” related to the local vegetation of given study area, and an ethological protocol for the animal species considered under study [93,94]. As a result, the DOM has been successfully implemented in different regions of México [102,103,104] and countries like France [93], Brazil [94], Greece [105] or Morocco [106].

The process of identification of plant species observed and collected has to be performed in specialized herbariums, and identification vouchers should be produced and preserved. There should always be a constant feedback between observers and botanists. The former supply the plant material for identification, which is performed by hand-plucking techniques [101], and the latter provide the specialized knowledge of plants using the correct keys for identification.

Another valuable tool for the initial screening of candidate plants could be derived from ethnoveterinary medicine. This discipline focuses on local ancient knowledge, beliefs, skills and practices concerning the prevention and cure of animal diseases, and continues to play an essential role in rural communities due to its accessibility and practicality [90,91]. The ethnoveterinary approach has been used to identify plants that might possess some activity against GIN [107,108,109,110]. It is worth mentioning the survey performed by Gradé et al. [110], which included 147 interviews with experienced shepherds or “healers” in Uganda and suggested the triggering of self-medicative behaviors in goats, naming them (in a curious manner) as *four-footed pharmacists*. In some ways, we believe that a nutraceutical feed, at some point, could result from performing scientific validations to plants selected using ethnoveterinary/ethnomedicine knowledge. Thus, the bridge between the ethnoveterinary and the validation of a nutraceutical potential might be the interdisciplinary approach that we are considering in the present review.

A third approach might be to consider plants and agricultural by-products that are known to contain certain types of PSC and which might serve as feedstuff for ruminant livestock. This is by far the most frequent approach used by different research groups all over the world. Different groups in Africa [111,112], Asia [113,114], Europe [115,116,117], Oceania [118], USA [119,120] or Latin-American countries such as Brazil [121,122], Colombia [123,124] and México [125,126], have searched their own CT-rich plant materials conducting extensive studies. This type of approach has also led to the discovery of bioactive agro-industrial by-products from coffee (*Coffea Arabica)* or cocoa (*Theobroma cacao)* [127,128] which will not be further considered in the present review as they are outside the use and management of LDF for small ruminant production.

### 3.3. The Agronomical Approach

A second step in the decision-process to assess the nutraceutical properties of plants should be assessing their availability. This factor can be an important constraint for the use of a nutraceutical material on-farm. In this subject, the decision-making process appears to be relatively simple:

- Plant species with a null or low intake by ruminants. These plants do not classify as potential nutraceuticals, but those with high availability could be considered for their use as phytotherapeutic or herbal remedies depending on subsequent PSC content analyses;
- Plant species consumed by ruminants but with low availability in the field. These are good prospects but have the constraint of not offering enough biomass for long-term consumption. This represents the main challenge for agronomists to increase the biomass production of promising candidates by means of improved agronomic practices or identification and the selection and breeding of high yielding varieties with good bioactivity;
- Plant species consumed by ruminants and with naturally good biomass availability in the field. These plants offer the best option to be considered as potential nutraceuticals.

Nonetheless, to determine the availability of plants in a heterogeneous environment is a labor-intensive process. Due to the nature of the LDF, biomass availability never remains constant, and therefore its assessment represents a critical factor. Farmers and shepherds manage LDF based on their assessment of biomass availability. However, few studies have formally assessed the biomass availability of LDF. Two recent studies covered this issue in Yucatán (southeast México), reaching descriptions at the plant species level and considering the edible material that can be effectively browsed by animals as observed under field conditions. The first study, by Ventura-Cordero et al. [129], implemented two randomly selected sites with different intervention degrees: site A (2.2 ha, pruned manually once every year) and site B (2.6 ha, left alone for years but previously used by Mayan populations for agricultural purposes). Assessment was performed in a 200 m^2^ (10 × 20 m) exclusion quadrant each site, where all the edible material was collected, identified and dried to quantify the biomass. In a second study, Torres-Fajardo et al. [130] employed thirty exclusion quadrants previously identified by Global Positioning System (2 × 2 m each) and collected all the edible material which were processed in the same way as the previous experiment. Salient results in the first experiment indicate that three plant species represented the 87.9% of availability in site A, while five plants accounted for 90.2% of availability in site B. In the second trial, trees, shrubs and herbs with high CT contents (>10%) made up slightly more than 70% of the availability. More detailed information specifying the availability of the different LDF plant species can be seen in Table 3.

Novel satellite based spectral methodologies like the normalized difference vegetation index, or the enhanced vegetation index could help to estimate biomass more simply and quickly, which could open new avenues of research [131]. Implementation of those techniques will need to be carefully calibrated using data obtained directly from the field to discriminate available biomass from edible available biomass.

### 3.4. The Nutritional Approach

Beyond the nutraceutical motivation, the evaluation of the nutritional value of feed resources available in different ecosystems should be a common practice in livestock farming. Unfortunately, this is not the case in most parts of the world. Broadly, the two options to assess the nutritional value of plant materials are based on in vitro and in vivo tests, although an ideal approach should include both of them. According to Hoste et al. [35], the plant species must be subjected to an information gathering process to assess: (i) the macronutrient profile, (ii) the digestibility and (iii) PSC and their possible harmful effects towards the animal species consuming them.

A conventional chemical and nutritional profile should include the assessment of parameters like crude protein (CP), metabolizable energy (ME), neutral detergent fiber (NDF), acid detergent fiber (ADF) and lignin. Digestibility is another important parameter when considering the nutritive value of ruminant feedstuffs. It is recommended that at least in vitro dry matter digestibility (IVDMD), in vitro organic matter digestibility (IVOMD), in vivo dry matter digestibility (DMD) and in vivo organic matter digestibility (OMD) be performed. A thorough compilation of macronutrient, fiber and in vitro digestibility of LDF plants has been produced [45,46,80]. It is expected that those feed resources show medium to high levels of CP and ME together with low levels of lignin and fibers. Furthermore, gathering information on the possible negative impact on health and production is also suggested. However, it is of great importance to consider the special features of the target small ruminant species. For instance, *Lantana camara* (Verbenaceae) presence in grazing systems has been warned due to the content of some pentacyclic triterpenoids (lantadenes) that may cause hepatotoxic effects [132]. The presence of alkaloids, saponins, and unknown volatile compounds affecting the nose, eyes and skin of people harvesting the leaves of different plant species from the LDF has been reported recently [133]. The hepatotoxic effect of this plant had been acquired from the cattle literature, and then extrapolated to small ruminants. However, Criollo goats readily consume patches of *L. camara* in the LDF, specially at the end of the rainy season [130] without evident negative side-effects. More recent reviews have highlighted the proteinic value of this plant for browsing ruminants [134].

### 3.5. The Secondary Compounds Assessment Approach

Another important issue in the search for nutraceutical plants is the determination of their PSC content. While macronutrients are ubiquitous in all plants, PSC have much more specified functions, making their identification more complex and their costs proportionally higher. As stated in the previous chapter, an enormous variety of PSC (around 200,000) occur in nature, with vast and versatile effects over different trophic levels. Broadly, the PSC are classified according to their chemical structures into phenolics, alkaloids, saponins and terpenes [51,52,53,135]. The present review focuses on the CT, which represents one in a myriad of PSC occurring in nature [29,30]. The CT, also known as proanthocyanidins, are the fourth largest group of PSC in the plant kingdom, belonging to the family of polyphenols [31] and are the most studied in livestock science. Broadly, these compounds possess a complex chemical structure with the basic ability to precipitate protein [135]. In spite of all the research conducted to unravel their nature and effects in ruminant nutrition and health, there are still large gaps of knowledge that continue to challenge different scientific disciplines.

In the same way as the nutritional approach, it is expected that candidate plants do not have too much PSC/CT. This criterion could serve as an initial, but not as a definitive guide, as there are some plant species of the LDF (i.e., *Neomillspaughia emarginata* or *Gymnopodium floribundum*) with a high concentration of CT (37.5 and 33.8%, respectively) that can make a large contribution to the diets of browsing sheep and goats [46,129,130]. Considering the latter, we want to emphasize two issues when targeting nutraceutical resources: (i) the relevance of identifying those readily consumed by animals as a first approximation (ethological approach); (ii) the fact that the quantity as well as quality of PSC are factors to consider in their bioactivity, and hence in their nutraceutical potential.

In order to perform reliable assessments of the CT content of target plant species, some aspects must be considered. Since CT are found in the cell walls and vacuoles of edible materials, including the stem, leaf, flower and seed of plants [136], tissue-specific localization in plant parts can vary widely, making it challenging to decide which of these structures will be subjected to analysis [30]. In addition, differences in the CT content of plants, even from the same geographic region (like LDF), were reported [137]. A large number of studies evaluating plants with AH activity have quantified the CT content in foliage, although this decision will depend on observational data (investing only in those resources consumed by small ruminants, i.e., flowers, bark, stem and pods), as well as the cost and availability of detection techniques. Another factor to consider is the influence of the drying protocol on the CT content of different plant materials [138,139]. On this subject, the general recommendation is to establish drying temperatures below 50 °C to avoid alterations in the CT content.

The chemical synthesis of CTs is quite complex, which means there are a multitude of CTs in existence [140]. In their elegant review about the structure, concentration and other chemical features of CTs, Mueller-Harvey et al. [31] addressed some interesting points related to this subject: (i) CT synthesis follows distinct biosynthetic patterns, (ii) the latter is described in terms of mean degree of polymerization (mDP), (iii) there are also procyanidin (PCs-type), prodelphinidin (PDs-type) and galloylated CTs, (iv) PCs-type are more widespread than PDs-type (v), many more plant species contain different PCs/PDs mixtures, and (vi) these structural properties are correlated with their bioactivity. Considering the latter, a detailed chemical characterization of the CTs is required to elucidate their mechanisms of action both in the nutrition and health of small ruminants.

Therefore, a comprehensive “*secondary compounds assessment approach*” should not focus its attention solely on the PSC contents. It is worth finding methods that may help to target the right part of the plant, and then store and process them properly so that they can be analyzed with the most reliable method.

### 3.6. The Parasitological (Anthelmintic) Approach

Once a plant material is confirmed to be consumed by small ruminants and to possess acceptable availability in the field, in addition to a good macronutrient content and PSC with possible bioactivity, then that plant material should undergo a series of tests in order to confirm its nutraceutical potential. To be consistent with the nutraceutical concept that was previously established [35], endeavors should be directed to evaluating the effect of that plant material on a relevant health condition. One of the models that has been extensively studied is the parasitic gastroenteritis caused by different species of GIN. Several studies attempted to identify feed resources capable to affect the GIN biology beyond their nutritional properties. The identification of plants affecting GIN could contribute to addressing the overwhelming challenge of AH-resistant GIN populations affecting the efficacy of commonly available drugs [141,142,143], or even the newest molecules [144,145]. Furthermore, society is currently demanding limits on the use of xenobiotics that are polluting animal products and the environment. Furthermore, GIN are one of the major challenges for sheep and goat production worldwide. This has special relevance for smallholders with limited access to technical advice (veterinary services) or who are confronted with economic limitations. Thus, the control of GIN using nutraceutical plants could become an easy-to-adopt practice, as farmers are very aware of and must frequently deal with the negative consequences of GIN parasitism.

#### 3.6.1. In Vitro Screening

The first parasitological approach in the identification process of a nutraceutical plant may be based on in vitro tests. These tests have some advantages over in vivo studies as they represent lower cost, rapid turnover and the possibility to screen a large number of plant materials in a short period of time. The procedure used for the evaluation of AH in the egg stage was adapted from the world association for the advancement of veterinary parasitology (WAAVP) guidelines to determine the efficiency of conventional synthetic AH drugs [146]. However, there is an urgency to adapt those guidelines to evaluate the complexity of PSC from plants. It is important to highlight that in vitro screening is based in the use of extracts obtained from different relevant parts of plants using different solvents in order to separate each group of PSC from the rest of the plant constituents. These types of crude extract still contain a number of different compounds, which will later require a partition process. However, this subject is beyond the scope of the present review. Another option would be to employ commercially available compounds which could be used to confirm or provide a first glimpse of the structure–activity relationship between compounds and nematodes. However, it should be remembered that, without a macronutrient supply, a plant cannot be considered as a nutraceutical. Thus, in vitro screening is necessary and provides key information in the process of identifying the nutraceutical potential of plants but cannot be seen as a definitive step.

**Figure 1:**
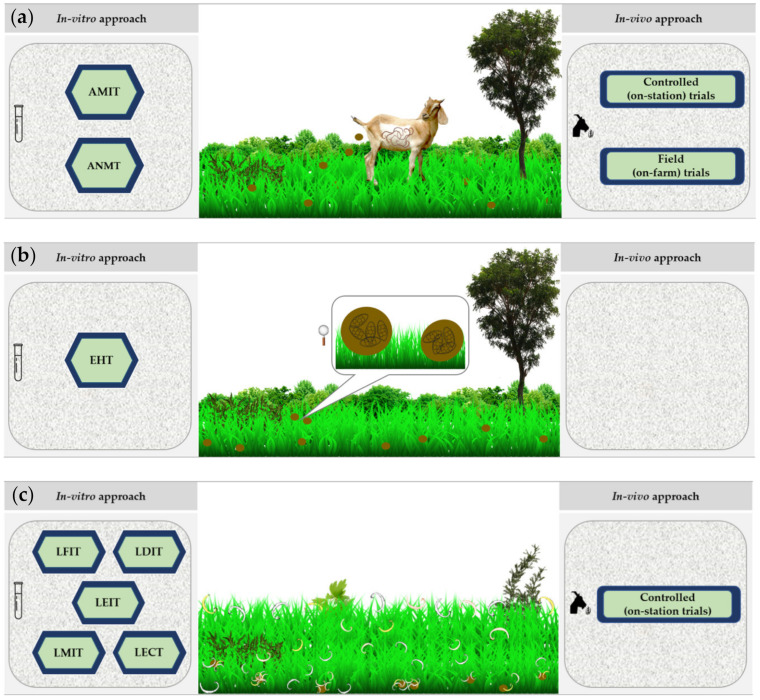
Biological cycle of gastrointenstinal nemotodes (GIN) and the methodologies that can be adopted to evaluate the nutraceutical potential of plants or plant extracts under laboratory or animal conditions. (**a**) Infected animal shed GIN eggs via feces. At this point, animals can be fed with the potentially bioactive plant(s) (controlled (on-station) trial) or can be taken to a natural feeding scenario (field (on-farm) trial). In addition, animals could be euthanized to obtain adult nematodes from different sections of the gastrointestinal system to perform the Adult Motility Inhibition Test (AMIT) and Adult Nematode Measurement Test (ANMT). Feces could be recollected to obtain, under laboratory conditions, eggs and larvae for subsequent controlled tests, (**b**) Feces contain GIN eggs that, under optimal environmental conditions, hatch in 1 or 2 days. Eggs can be used for the Egg Hatch Test (EHT). (**c**) The larvae, influenced by environmental factors, undergoes three moults until they reach the infective stage (L_3_). The larvae can be used for the Larvae Feeding Inhibition Test (LFIT), Larvae Development Inhibition Test (LDIT), Larvae Exsheathment Inhibition Test (LEIT), Larvae Motility Inhibition Test (LMIT) and Larvae Establishment Capacity Test (LECT). In addition, short-term feeding trials might be conducted at the same time of artificial GIN infection to evaluate the effect of plant materials in the establishment of parasites.

The current in vitro tests are focused on assessing the effect of extracts against specific stages of the biological cycle of GIN, broadly divided into free-living (eggs and larvae) and parasitic (adults) stages. The techniques, materials and specifications were thoroughly addressed by Bauhaud et al. [147] and Jackson and Hoste [148]. Some of the methodologies used to evaluate the in vitro AH activity of plant extracts include the egg hatching test (EHT), larval feeding inhibition test (LFIT), larval development inhibition test (LDIT), larval exsheathment inhibition test (LEIT), larval motility inhibition test (LMIT), adult motility inhibition test (AMIT) and adult nematode measurement test (ANMT). Recently, a study implemented the larval establishment capacity test (LECT) using ethanolic extracts of *Artemisia cina* [149]. A schematization of the biological/epidemiological cycle of GIN and the possibilities for the in vitro and in vivo parasitological approach on each stage are presented in Figure 1.

Given that in vitro tests are directed to various life stages of GIN (morulated and embryonated eggs, 3rd and 4th larval stages and adult nematodes), some considerations should be taken to avoid generalizations. At each stage, complex metabolic and chemical processes occur. Thus, obtaining an AH effect on a specific stage (i.e., eggs), cannot be extrapolated to another stage (i.e., the infective L_3_ or the adults). In addition, the process used to obtain the extract could significantly affect the expression and isolation of different PSC. When the CT are the targeted PSC, extraction with acetone/water (70:30) or methanol/water (70:30) represents the most common and efficient solvents in regard to yield of extraction [35]. When the chemical structure of CT is related to the in vitro AH activity, it has been found that: (i) high molar percentages of PD-type and galloylation showed AH effects against GIN larvae, (ii) high values of mDP also showed AH activity, and (iii) galloylated CT showed particularly antioxidant, antimicrobial and nematocidal activity but are represent a poorly studied group [31,150]. Effective concentrations of 50%, 90% and 99% (EC_50_, EC_90_ and EC_99_, respectively) are parameters used for assessing or comparing the in vitro bioactivity of many extract partitions or chemical compounds. However, there is a lack of well-established thresholds to consider AH activity against biological stages of ruminants’ GIN [151]. Based on the experiences of many tests conducted with LDF plants, we think that an EC_50_ value of 1000 µg/mL could be consider as the highest threshold to be considered good AH bioactivity.

#### 3.6.2. In Vivo Screening

In spite of the relevance of in vitro tests in the screening process of plants, they do not deal with the interference of the multifactorial environment inside the ruminant hosts. The main discrepancies between in vitro tests and in vivo trials are related to the static vs. dynamic systems in which these procedures are conducted, which includes the need to reach a certain level of the PSC in the lumen of the gastrointestinal tract, with the need to reach a high intake of the plant material to allow an effective concentration under in vivo conditions. It is important to consider the ruminal adaptations and changes in microbial populations and the rate and sequence at which PSC enter and are processed in the digestive tract [140]. Thus, due to the invariable difficulty to duplicate those factors, testing in the host is the best way to determine the true nutraceutical potential of plant materials [152]. The in vivo approach is focused on the use of two experimental contexts with different fundamentals and methodologies. First, the controlled/on-station approach, which is based on the administration of one or various plants to animals in a supervised scenario and second, the natural condition or field/on-farm approach, which evaluates the feeding decisions of animals when they are allowed to eat freely in heterogenous vegetation scenarios.

**Figure 2:**
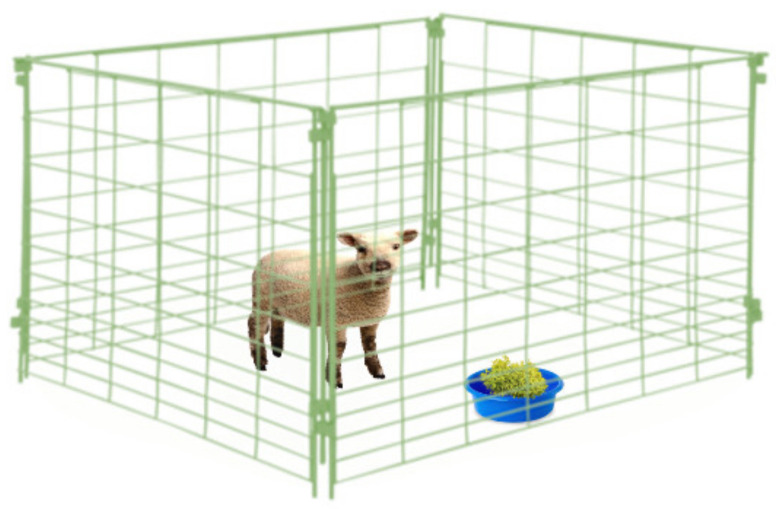
On-station trial for the evaluation of in vivo anthelmintic properties of plants. When one plant species is under evaluation, the voluntary feed intake might be related with the dynamics of GIN infection. Nutritional and immunological aspects should be also considered. Some researchers choose to hang experimental plants to simulate browsing ingestive behaviors. Such considerations should be the function of the facilities where trials are conducted.

**Figure 3:**
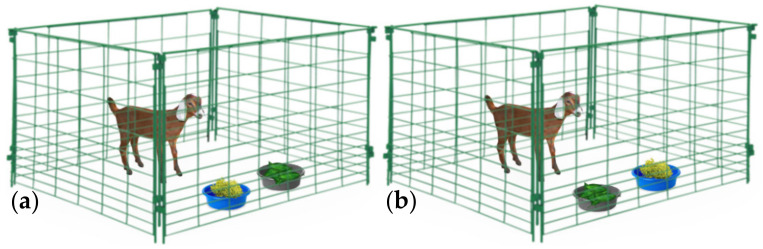
On-station trial for the evaluation of in vivo anthelmintic properties of plants. When a cafeteria trial of two plant species are performed, it is recommended that the candidate for nutraceutical properties contains a known quantity of a plant secondary compound while the other plant does not. (**a**,**b**) represent the first and second experimental days, respectively. For the establishment of control groups, a polymer (polyethylene glycol) could be used to neutralize condensed tannins and other polyphenols. Activated charcoal could be used to neutralize some terpenes. Containers in which the plants are offered must be equidistant and changed on a daily basis to avoid associate learning. Some researchers choose to hang experimental plants to simulate browsing ingestive behaviors. Such considerations should be in function of the facilities where trials are conducted.

**Figure 4:**
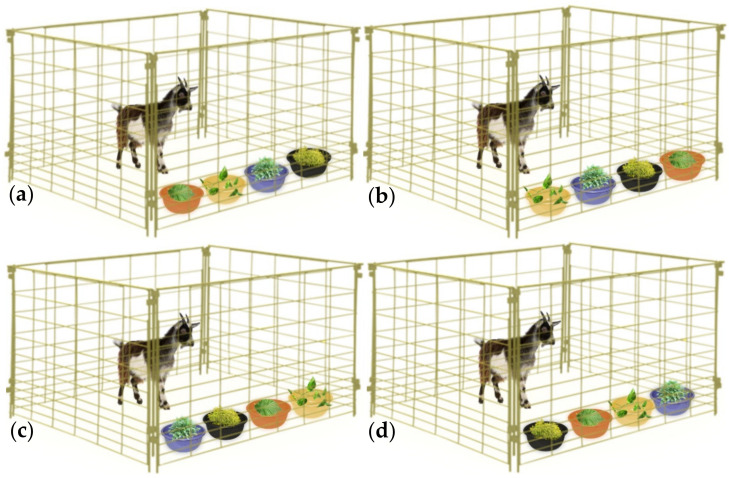
On-station trial for the evaluation of in-vivo anthelmintic properties of plants. Usage of more than two plant species implies a more realistic scenario albeit bioactivity of a specific plant should be considered cautiously. (**a**–**d**) represent the first, second, third and fourth experimental days. Containers in which the plants are offered must be equidistant and changed on a daily basis to avoid associate learning. Some researchers choose to hang experimental plants to simulate browsing ingestive behaviors. Such considerations should be in function of the facilities where trials are conducted.

When the AH activity of a plant material has been evidenced using in vitro tests, their nutraceutical value has to be validated by administering that plant to animals under controlled conditions, and thus establishing the relationship between its voluntary feed intake (VFI) and the GIN infection. Two scenarios could be adopted in order to elucidate such nutraceutical properties: (i) administer the single elected plant to the animals during a determined period or, (ii) a slightly more realistic scenario, which includes the administration of many plants (two or more) offered at the same time, thereby simulating natural feeding scenarios, which has been named a ‘preference test’, ‘preference study’, ‘preference trial’, ‘palatability experiment’, ‘cafeteria trial’, ‘cafeteria method’ or ‘cafeteria technique’ [153]. Both on-station methodologies (single plant assessment and cafeteria trial) provide the possibility of assessing factors influencing the animal’s decision of what and how much plant material to consume [153,154]. That may allow researchers to understand the ingestive choices of animals under complex scenarios and may serve as a component in the screening process of plants with nutraceutical potential. A proper implementation of cafeteria trials in a two-plant choice should consider that the additional plant did not have contents of the PSC with potential AH activity (serving as a control), whereas, in the case of a three-or-more plants trial, caution should be taken when declaring a direct AH effect of specific plants due to the mixed ration that the animals are consuming. Then, previous knowledge of nutritional and PSC content, as well as precise intake assessment of experimental plants on a daily basis, are needed to suggest specific plant bioactivity. Considering the macronutrient content of the tested plant material(s), emphasis should be placed on administering a diet with known protein and energy contents to experimental animals in order to avoid any nutritional confounding effect. Ignoring this issue would not allow researchers to differentiate between what might be considered as an “indirect nutritional effect” on the resilience and resistance against GIN, as opposed to the “direct effect” of PSC as a non-conventional AH. Interactions between the nutritional status of animals and expression of resilience and resistance to GIN infection development are well-known phenomena [155,156,157]. Hence, detailed knowledge of nutritional and PSC contents of feed resources is necessary to differentiate their nutritional and health effects, especially when animals are consuming a plant *mix*, for example in cafeteria trials or a natural scenario such as LDF. It is highly recommended to implement an adaptation/familiarization period of experimental animals with the conditions and the plant material that need to be administered. The latter can consider behavioral issues and to allow for ruminal environment adaptations [153]. During the in vivo controlled trials with two or more plant materials, the position of plants should be changed daily to avoid any bias related with learning processes [158]. The nature of GIN infection is also a factor to keep in mind, although, considering the controlled approach, an artificial infection with one or more GIN species is the only acceptable option, hence the production of specific GIN strains is necessary [159]. A synthesis of some procedures and considerations of the controlled/on-station trials are shown in Figure 2, Figure 3 and Figure 4.

The field or on-farm approach challenge animals with the highly varied and variable feed offer the characteristics of rangelands in a similar fashion, as they are maintained by farmers in production systems where they use/manage the LDF for grazing/browsing. As stated by Agreil et al. [96], this approach aims to the ‘naturalization’ of objects under study, instead of trying to isolate the world under observation to manipulate selected processes. Hence, trials under natural scenarios allow us to understand the feeding strategies of animals at various scales together with the identification of plants with nutraceutical potential. In this context, the cornerstone of these studies lies in the adoption of a methodology which allows researchers to monitor and quantify the feeding behavior of animals. As previously mentioned, there are indeed ethologic methodologies based on continuous observations of domestic herbivores within their grazing circuits (e.g., DOM), which have been widely used [91,92]. During the execution of these trials, some aspects are recommended: (i) adult animals with previous knowledge of the wide plant array are preferred over unexperienced animals. The former have indeed a well-established *grazing culture* while the latter do not, (ii) the grazing circuit, and their duration should ideally be maintained before and during each trial, (iii) natural GIN infection would be the best choice, (iv) a temporal scale must be defined (i.e., hours, days, weeks, seasons) and (v) given the dynamic nature of the approach, monitoring of GIN burden and/or physiological parameters should be ideally considered over the entire protocol.

Focusing on the CT, an important fact must be considered. Given the relevance of establishing control groups in experimental designs, together with the large number of PSC contained in plant materials, it is difficult to find two similar species that allow for effective comparisons. Then, neutralizing the specific PSC of the plant material offers a strategy to circumvent this constraint. It is generally recognized that two compounds called polyvinyl pyrrolidone (PVPP) and polyethylene glycol (PEG) are able to block/neutralize the CT due to their high affinity for these PSC. The PVPP have been used more for the in vitro tests, while PEG is more recommended for in vivo trials. The reader is referred to [25,30,56,160,161] for a detailed description of the role of CT-neutralizing polymers in small ruminant nutrition.

A needful reminder is that conclusions derived from this point should be taken cautiously. Finding no AH effect of a plant under the in vitro and/or in vivo approaches does not necessarily classify that plant as non-nutraceutical. One has to bear in mind is that our approach is directed to unravel nutraceutical properties of plants using GIN infection as a model, nonetheless, other positive effects on ruminant health, including aspects such as anti-bloating, antiviral, antimicrobial, antiprotozoal and antifungals, among others [84,85,86,87], may represent other important avenues for the investigation of their nutraceutical potential. A clear example of this is the ethnoveterinary knowledge which farmers have traditionally used to treat different illnesses in addition to GIN infections.

### 3.7. The Animal Approach

The last approach in the identification process of nutraceutical plants is to consider which small ruminant species will be proposed for the in vivo trials. The traditional and cultural value of sheep and goats worldwide, especially in developing countries, has considered them with the misconceived view that both species are the same. Hence, the knowledge acquired from one is usually extrapolated to the other, which has brought negative consequences for the health and production of these livestock species. Nowadays, it is well known that sheep and goats have differences in several aspects, amongst them, parasitological, behavioral, immunological and pharmacological features that will be briefly considered below.

A common factor between sheep and goats relies on the species of helminths affecting them, which includes trematodes and cestodes, as well as GIN [23,58,59]. However, the size of parasite populations can be very different even for species simultaneously consuming the same pastures [60,71]. Experience acquired from sheep had been assumed to be applicable for goats, which has contributed to pharmacological and posology mistakes, resulting in a threatening increase in GIN-resistant strains in goats [162]. It is noteworthy to remember that the pharmacodynamics of AH drugs in goats is faster that in sheep, leading to a general and well-accepted notion of establishing specie-specific dosages, with goats receiving 1.5 to 2 times the doses administered in sheep [163,164].

Shrubs and grasses are two functionally and ecologically distinct components of terrestrial vegetation [165], differing in their morphology, chemistry, 2d/3d structure and distribution. The latter has led to morphological, physiological and behavioral adaptations of herbivores that feed on these type of resources [166]. For a thorough understanding of these morphological and physiological adaptations, the reader is referred to several elegant reviews [21,56,165,166,167].

Behaviorally speaking, the classification of herbivores according to their innate dietary preferences [168] considers goats, which rely mainly on the consumption of shrubs, vines and herbs, as intermediate browsers, while sheep, which rely mainly on the consumption of grass species are classified as grazers. However, some authors argue that sheep are also intermediate feeders [169]. Despite that, it is well recognized that goats possess a higher ability to adapt to harsh environments or unfavorable conditions, being able to select and consume higher quantities of browse and PSC compared with any other domesticated ruminants [170,171,172,173]. These behavioral features have important implications for the control of GIN infection through nutraceutical plants.

Small ruminant species use different behavioral approaches to cope with the burdens of the GIN present in the pasture, namely “*fight*” or “*flight*” behaviors [174]. Sheep seem to rely more on the “*fight*” strategy since they have longer exposure times, with the infective L_3_ present in low stratum feedstuffs such as grasses. The latter implies the existence of a better sheep immune response against GIN than is evident for goats, influencing the establishment, development, growth, fertility and the production of different GIN stages [175,176]. On the other hand, goats, as intermediate feeders, ingest more browse/shrub material where the presence of L_3_ is low or null, hence they adopt the “*flight*” strategy, as will be discussed below. However, this might imply a lower expression of the immune responses against GIN in goats compared to sheep [174], but a faster xenobiotic (PSC and AH) metabolism [163,176] and a higher propensity to self-medicate [110,174,177]. Nevertheless, a detailed discussion of the species approach is beyond the scope of this review.

Finally, it is worth remembering that the findings and conclusions derived from the different methodological tools developed to evaluate nutraceuticals cannot be extrapolated to other livestock species (e.g., cattle or pigs). Thus, specification of the experimental context with evaluated and adapted methods for any particular animal and plant species, laboratory procedures, quantitative/qualitative assays and laboratory/field conditions leads to more precise and convenient discoveries.

### 3.8. The Selectivity Assessment Approach

We think that selection could be considered as a novel criterion that could fit properly within the interdisciplinary approach. Then, this concept needs to be clarified and differentiated from related terms such as intake and preference: (i) intake is the quantity of dry matter consumed by an animal in a determined time period [178,179], (ii) preference is the likelihood that a resource will be selected if offered on an equal basis with other resources [180] and, (iii) selection is a multifactorial process in which animals choose feed resources disproportionately to the plant availability [181]. To assess selection, it is necessary to employ selectivity indexes, which are mathematic relationships between the proportions of used and available resource items [182]. Incorporating selection indexes in the screening process for nutraceutical plants could be useful to understand the degree at which the plant resources are used, as well as the herbivory pressure for specific plants, especially under natural feed conditions where resources’ availability is an ever-changing process. However, biomass assessment (a requisite for the estimation of selection indexes) in heterogeneous systems is a hard-and labor-intensive procedure that requires methodological, agronomical and environmental considerations to be properly performed. It is worth mentioning that selection can be assessed under controlled cafeteria trials by using specific selection indexes and ensuring a constant supply of the feed resources [183,184]. An estimation of selectivity has been performed under natural [129,130] and controlled [185,186,187] conditions in the LDF and other ecosystems [105,106,186,187].

## 4. Final Remarks and Implications

In the present review, the particular context of the LDF was presented, the concept of the term nutraceutical was established and, an interdisciplinary approach to the screening process of the nutraceutical properties of plants was addressed. The knowledge of the nutritional and therapeutic value of different plant species of the LDF could help in the design of management strategies that allow for improving animal and farm productivity, animal health and, consequently, human health and well-being through the consumption/marketing of its products.

To date, approximately 80,000 plants are suggested to exert some influence on animal health and productivity [188]. However, it is likely that there are a vast number of nutraceutical feed resources waiting to be identified in different native vegetation systems worldwide. Zeineldin et al. [189] mentions that a web-based search using the words “*bioactive plants as anthelmintic*” yields over 1000 citations. Undoubtedly the more-studied PSC in terms of ruminant nutrition and AH activity are the CT [13,27,28,29,30]. Nonetheless, it is known that other PSC such as flavonoids, saponins, terpenes, alkaloids, hydrolysable tannins, among others, could influence the physiology of both ruminants and nematodes [34,190,191,192,193,194,195,196]. Hence, considering the plethora of plant species and PSC present in the LDF, we have a wide avenue of future research. Considerable insight has been gained, although there are novel factors to account for, as we only have a glimpse of the whole picture.

The majority of studies related to the assessment of nutraceutical properties of plants have been performed using in vitro tests [34,35], which could be related to their lower cost, rapid turnover and the possibility of obtaining and testing the activity of specific PSC through the use of solvents [148,152]. On the contrary, during in vivo trials, intake of different levels and concentrations of both nutrients and PSC is unavoidable when experimental plants are consumed (the higher the number of plants, the harder the possibility of interpreting the results). Then, the administration of PSC-neutralizing agents was proposed to establish control groups and then distinguish bioactive effects. To the best of our knowledge, only a limited number of PSC-neutralizers have been identified. Charcoal has been reported to neutralize terpenes [197,198], while PVPP and PEG have been reported to neutralize polyphenols like CT and flavonoids [160,161]. There is a necessity to further studies focusing on the identification of chemicals capable of neutralizing the other classes of PSC.

The tree species in the LDF can reach heights up to 15 m [39]; as a consequence, there is a constraint for small ruminants to consume their foliage. Indeed, when measuring availability, biomass found above 2 m was not considered since those trees, in most instances, cannot be physically reached by small ruminants. Thus, the mean percentage of biomass availability of Fabaceae trees like *H. albicans*, *A. pennatula*, *M. bahamensis*, *L. leucocephala*, *S. gaumeri*, *A. collinsi*, *B. divaricata* and *L. latisiliquum* was 4.0, 1.7, 1.7, 1.0, 0.75, 0.2, 0.1 and <0.1, respectively (Table 3). Nonetheless, the net biomass yield of the entire tree is likely to be higher. Cut-and-carry systems could represent a viable alternative to guarantee that animals are exposed constantly to potential nutraceutical material that is difficult to obtain within their feeding circuits [35]. However, its feasibility (labor, time and transportation) must be studied before on-farm implementation is suggested.

**Figure 5:**
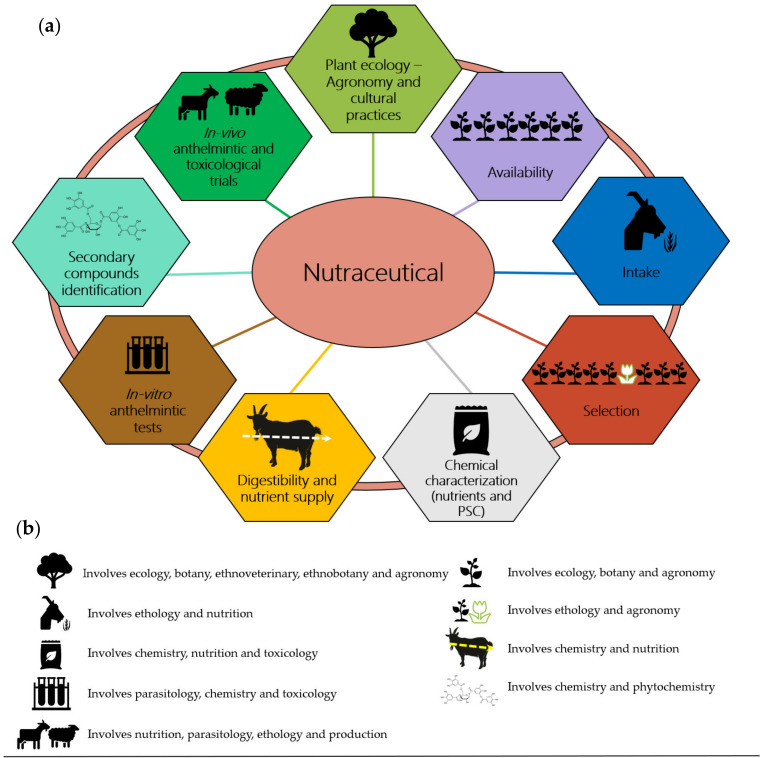
Proposed protocol for the identification of plants with nutraceutical potential. (**a**) For its proper implementation, an interdisciplinary approach must be developed considering knowledge from different scientific disciplines. It is worth considering that every component under consideration feedbacks the other in a cyclic manner. Thus, investigation of nutraceuticals in livestock must be considered as a process instead an isolated event. (**b**) Every criterion is related to one or various knowledge disciplines, which implies that evaluation of the nutraceutical nature of plants is derived from interdisciplinary endeavors.

Finally, in order to schematize some of the criteria previously addressed to screen plant materials, we present Figure 5a,b, which highlights the proposal protocol for the identification of plants with nutraceutical potential and the main disciplines related to this process.

## 5. Conclusions

There is a need for the standardization of guidelines for the scientific evaluation of the nutraceutical properties of plant species, similar to those developed to assess the efficacy of synthetic AH drugs against ruminants’ GIN. There are still many knowledge gaps and plenty of endeavors to be performed. However, we hope this review may contribute to the conceptualization and construction of future official guidelines. The knowledge and use of plants from the LDF, and by extension from other ecosystems, should help in the design of sustainable production systems with an improved use of native resources, reducing the use of external xenobiotics and, as a result, reducing their environmental impact. 

## Figures and Tables

**Table 1:** Definitions related to feed characteristics commonly associated to the nutraceutical concept.

Concept	Definition	Comment	Reference
Feed additive	A compound generally of synthetic origin added to the feed	Dependent of VFI and long-term administration to exert their effects	[35,86,89]
Cosmeceutical	A cosmetic product with active ingredients	Independent of VFI. Medium to long-term administration to exert their effects	[78,88]
Functional food	A food containing nutrients and ingredient(s) with beneficial effects beyond adequate nutritional properties	Dependent of VFI and need long-term administration to exert their effects. The closest to the nutraceutical definition, however functional food includes fortified foods while nutraceuticals do not	[85,86]
Phytotherapeutic	A compound of natural origin. It can be a plant, or its extracts used instead of “conventional drugs”	Independent of VFI and need short-term administration to exert their effects	[35,85]
Ethnoveterinary or ethnomedicine	Practices derived from ancient knowledge, beliefs and skills to prevent and treat animal diseases	Dependent of VFI and need long-term administration to exert their effects	[90,91]

VFI: Voluntary feed intake.

**Table 2:** The evolution of the nutraceutical concept for non-ruminants and ruminants over time.

Definition	Year	Reference
Food or part of a food that provides health benefits and is used for prevention or treatment of a disease	1989	[82,83]
A nondrug substance that is produced in a purified or extracted form and administered orally to provide agents required for normal body structure and function with the intent of improving the health and wellbeing of animals	1997	[91]
Any substance that may be considered a food or part of a food which provides health benefits, including the prevention and treatment of disease	2002	[92]
A livestock feed which combines nutritional value with beneficial effects on animal health	2015	[35]
Natural compounds and/or microbes that offer potentially advantageous effects to ruminant health and productivity, including improved efficiency, milk production, and disease resistance through immune modulation or decrease disease pressure	2019	[86]

**Table 3:** Availability of edible plant feed resources for small ruminants during the rainy season in the Low Deciduous Forest of Yucatán, México.

Plant Species	Family	Life Form	Availability (Kilograms of Dry Matter per Hectare)	%	Reference
***Acacia collinsi***	Fabaceae	Tree	4.8	0.2	[130]
***Acacia pennatula***	Fabaceae	Tree	37.6	2.5	[129]
			26.8	0.9	[130]
***Bahuinia divaricata***	Fabaceae	Shrub/Tree	2.6	0.1	[130]
***Caesalpinia gaumeri***	Fabaceae	Tree	15.3	1.0	[129]
			10.3	3.9	[129]
			183.8	6.2	[130]
***Caesalpinia yucatanensis***	Fabaceae	Shrub/Tree	1.7	0.1	[130]
***Chamaecrista glandulosa***	Fabaceae	Herb/Shrub	0.3	0.1	[129]
			1.7	0.1	[130]
***Diphysa yucatanensis***	Fabaceae	Shrub/Tree	5.7	0.2	[130]
***Havardia albicans***	Fabaceae	Tree	118.9	4.0	[130]
***Leucaena leucocephala***	Fabaceae	Shrub/Tree	15.6	1.0	[129]
			25.4	0.9	[130]
***Lysiloma latisiliquum***	Fabaceae	Tree	0.3	<0.1	[130]
***Mimosa bahamensis***	Fabaceae	Shrub/Tree	4.3	0.3	[129]
			0.9	0.3	[129]
			83.5	2.8	[130]
***Piscidia piscipula***	Fabaceae	Shrub/Tree	0.3	0.1	[129]
			6.7	0.2	[130]
***Senegalia gaumeri***	Fabaceae	Tree	16.3	0.5	[130]
***Senna villosa***	Fabaceae	Shrub/Tree	7.4	0.3	[130]
***Ipomoea crynicalix***	Convolvulaceae	Vine	119.7	8.0	[129]
			72.1	27.4	[129]
			115.3	3.9	[130]
***Ipomoea nil***	Convolvulaceae	Vine	1.2	0.1	[129]
			1.8	0.1	[130]
***Jaquemontia penthanta***	Convolvulaceae	Vine	0.3	<0.1	[130]
***Merremia aegyptia***	Convolvulaceae	Vine	5.0	1.9	[129]
			0.3	<0.1	[130]
***Helicteres baruensis***	Malvaceae	Shrub	2.5	0.9	[129]
***Sida acuta***	Malvaceae	Herb	1.7	0.1	[130]
***Waltheria indica***	Malvaceae	Herb	66.6	4.4	[129]
			10.7	4.1	[129]
			26.3	0.9	[130]
***Bidens pilosa***	Asteraceae	Herb	28.5	1.9	[129]
***Porophylum punctatum***	Asteraceae	Herb	3.4	0.2	[129]
			3.5	0.1	[130]
***Viguiera dentata***	Asteraceae	Herb	5.3	2	[129]
			3.8	0.1	[130]
***Gymnopodium floribundum***	Polygonaceae	Shrub/Tree	1132.8	75.5	[129]
			129.7	49.3	[129]
			1653.8	55.5	[130]
***Neomillspaughia emarginata***	Polygonaceae	Shrub/Tree	16.3	1.1	[129]
			66.3	2.2	[129]
***Podopterus mexicanus***	Polygonaceae	Shrub/Tree	60.6	2	[130]
***Morinda royoc***	Rubiaceae	Vine	1.8	0.1	[130]
***Randia aculeata***	Rubiaceae	Shrub	2.8	0.2	[129]
			4.3	0.1	[130]
***Randia obcordata***	Rubiaceae	Shrub/Tree	94.9	3.2	[130]
***Cordia alliodora***	Boraginaceae	Tree	30.7	1	[130]
***Cordia globosa boroginosa***	Boraginaceae	Shrub/Tree	35.8	2.4	[129]
***Stachytarpheta jamaicensis***	Verbenaceae	Shrub	0.4	<0.1	[130]
***Lantana camara***	Verbenaceae	Shrub	3	0.1	[130]
***Acalypha*** **spp.**	Euphorbiaceae	Herb	9.6	3.6	[129]
***Cnidoscolus aconitifolius***	Euphorbiaceae	Shrub/Tree	3.4	<0.1	[130]
***Tetramerium nervosum***	Acanthaceae	Herb	0.3	<0.1	[130]
***Parmentiera millspaughiana***	Bignoniaceae	Shrub/Tree	2.4	0.1	[130]
***Bursera simaruba***	Burseraceae	Tree	2	0.1	[130]
***Dioscorea polygonoides***	Dioscoraceae	Vine	2.1	0.8	[129]
			0.3	<0.1	[130]
***Diospyros anisandra***	Ebenaceae	Shrub/Tree	65.6	2.2	[130]
***Mentha villosa***	Lamiaceae		15.2	0.5	[130]
***Bunchosia swartziana***	Malpighiaceae	Shrub/Tree	15.4	0.5	[130]
***Passiflora biflora***	Passifloraceae	Herb	1.8	0.1	[130]
***Eragrostis ciliaris***	Poaceae	Grass	15.3	1	[129]
			14.5	5.5	[129]
			283.8	9.5	[130]
***Karwinskia humbdoltiana***	Rhamnaceae	Tree	5.8	0.4	[129]
***Cardiospermum corindum***	Sapindaceae	Vine	1.5	0.1	[130]
***Alvaradoa amorphoides***	Simaroubaceae	Shrub/Tree	23	0.8	[130]
***Solanum tridynamum***	Solanaceae	Shrub	4.8	0.2	[130]

[129] Used a single exclusion quadrant of 200 m^2^ (20 × 10 m), [130] Used 30 exclusion quadrants (2 m × 2 m), <0.1 Corresponds to those plant species whose availability in the field was less than 0.1% of the total biomass availability.

## References

[1] Estell R.E., Havstad K.M., Cibilis A.F., Fredrickson E.L., Anderson D.M., Schrader T.S., James D.K. (2012). Increasing shrub use by livestock in a world with less grass. Rangel. Ecol. Manag..

[2] McDermott J.J., Staal S.J., Freeman H.A., Herrero M., Van de Steeg J.A. (2010). Sustaining intensification of smallholder livestock systems in the tropics. Livest. Sci..

[3] Flores J., Bautista F. (2012). Knowledge of the Yucatec Maya in seasonal tropical forest management. Rev. Mex. Biodivers..

[4] Accatino F., Sabatier R., De Michele C., Ward D., Wiegand K., Meyer K.M. (2014). Robustness and management adaptability in tropical rangelands: A viability-based assessment under the non-equilibrium paradigm. Animal.

[5] Lund H.G. (2007). Accounting for the world’s rangelands. Rangelands.

[6] Alonso-Díaz M.A., Torres-Acosta J.F.J., Sandoval-Castro C.A., Campbell W.B., Bruce Campbell W., López-Ortíz S. (2014). Controlling the Introduction and Augmentation of Parasites in and on Domesticated Livestock. Sustainable Food Production Includes Human and Environmental Health.

[7] Dumanski J., Desjardins R.L., Lal R., Rosegrant M., de Freitas L., Lander J.N., Gerber P., Steinfeld H., Verchot L.V., Schuman G.E., Stitger K. (2010). Supporting evidence for greenhouse gas mitigation in agriculture. Applied Agrometeorology.

[8] Ayatunde A., de Leeuw J., Turner M.D., Said M. (2011). Challenges of assessing the sustainability of (agro)-pastoral systems. Livest. Sci..

[9] Oosting S.J., Udo H.M.J., Viets T.C. (2014). Development of livestock production in the tropics: Farm and farmers’ perspectives. Animal..

[10] Hubert B., Meuret M., Bonnemaire J., Hadorn G.H., Hoffmann-Riem H., Biber-Klemm S., Grossenbacher-Mansuy W., Joye D., Pohl C., Wiesmann U., Zemp E. (2008). Shepherds, sheep and forest fires: A reconception of grazinglands. Handbook of Transdisciplinary Research.

[11] Hubert B., Deverre C., Meuret M., Meuret M., Provenza F.D. (2014). The rangelands of southern France: Two centuries of radical change. The Art and Science of Shepherding.

[12] Udo H.M.J., Aklilu H.A., Phong L.T., Bosma R.H., Budisatria I.G.S., Patil B.R., Samdup T., Bebe B.O. (2011). Impact of intensification of different types of livestock production in smallholder crop-livestock systems. Livest. Sci..

[13] Torres-Acosta J.F.J., Alonso-Díaz M., Hoste H., Sandoval-Castro C.A., Aguilar-Caballero A.J. (2008). Positive and negative effects in goat production arising from the intake of tannin rich forage. Trop. Subtrop. Agroecosyst..

[14] Torres-Acosta J.F.J., González-Pech P.G., Ortíz-Ocampo G.I., Rodríguez-Vivas R.I., Tun-Garrido J., Ventura-Cordero J., Castañeda-Ramírez G.S., Hernández-Bolio G.I., Sandoval-Castro C.A., Ortega-Pacheco A. (2016). Revalorizando el uso de la selva baja caducifolia para la producción de rumiantes. Trop. Subtrop. Agroecosyst..

[15] Ventura-Cordero J., Sandoval-Castro C.A., González-Pech P.G., Torres-Acosta J.F.J. (2017). El follaje de la selva baja caducifolia como alimento nutracéutico y su potencial antihelmíntico en pequeños rumiantes. AIA.

[16] Flores J., Vermont R., Kantún J., Sandoval-Castro C.A., Deb Hovell F.V., Torres-Acosta J.F.J., Ayala-Burgos A. (2006). Leguminosae diversity in the Yucatán peninsula and its importance for sheep and goat production. Herbivores: The Assessment of Intake, Digestibility and the Roles of Secondary Compounds.

[17] Mithofer A., Boland W. (2012). Plant Defense Against Herbivores: Chemical Aspects. Ann. Rev..

[18] Moore B.D., Wiggins N.L., Marsh K.J., Dearing M.D., Foley W.J. (2015). Translating physiological signals to changes in feeding behavior in mammals and the future effects of global climate change. Anim. Prod. Sci..

[19] Janis C., Gordon I.J., Prins H.H.T. (2008). An evolutionary history of browsing and grazing ungulates. The Ecology of Browsing and Grazing.

[20] Clauss M., Kaiser T., Hummel J., Gordon I.J., Prins H.H.T. (2008). The morphophysiological adaptations of browsing and grazing mammals. The Ecology of Browsing and Grazing.

[21] Estell R. (2010). Coping with shrub secondary metabolites by ruminants. Small Rum. Res..

[22] Molento M.B., Fortes F.S., Pondelek D.A.S., Borges F.A., Chagas A.C.S., Torres-Acosta J.F.J., Geldfoh P. (2011). Challenges of nematode control in ruminants: Focus on Latin America. Vet. Parasitol..

[23] Miller J.E., Kaplan R.M., Pugh D.G., Pugh D.G., Baird A.N. (2012). Internal parasites. Sheep and Goat Medicine.

[24] Charlier J., van der Voort M., Kenyon F., Skuce P., Vercruysse J. (2014). Chasing helminths and their economic impact on farmed ruminants. Trend. Parasitol..

[25] Makkar H.P. (2003). Effects and fate of tannins in ruminant animals, adaptation to tannins, and strategies to overcome detrimental effects of feeding tannin-rich feeds. Small Rum. Res..

[26] Mueller-Harvey I. (2006). Unravelling the conundrum of tannins in animal nutrition and health. J. Sci. Food Agric..

[27] Alonso-Díaz M.A., Torres-Acosta J.F.J., Sandoval-Castro C.A., Hoste H. (2010). Tannins in tropical tree fodders fed to small ruminants: A friendly foe?. Small Rum. Res..

[28] Hoste H., Martínez-Ortíz-de-Montellano C., Manoralaki F., Brunet S., Ojeda-Robertos N., Fourquaux I., Torres-Acosta J.F.J., Sandoval-Castro C.A. (2011). Direct and indirect effect of bioactive tannin-rich tropical and temperate legumes against nematode infections. Vet. Parasitol..

[29] Muir J. (2011). The multi-faceted role of condensed tannins in the goat ecosystem. Small Rumin. Res..

[30] Piluzza G., Sulas L., Bullita S. (2014). Tannins in forage plants and their role in animal husbandry and environmental sustainability: A review. Grass Forage Sci..

[31] Mueller-Harvey I., Bee G., Dohme-Meier F., Hoste H., Karonen M., Kölliker R., Lüscher A., Niderkorn V., Pellikaan W.F., Salminen J.P. (2019). Benefits of condensed tannins in forage legumes fed to ruminants: Importance of structure, concentration, and diet composition. Crop. Sci..

[32] Kamaraj C., Rahuman A., Elango G., Bagavan A., Zahir A.A. (2011). Anthelmintic activity of botanical extracts against sheep gastrointestinal nematodes, Haemonchus contortus. Parasitol. Res..

[33] Mravčáková D., Váradyová Z., Kopčáková A., Čobanová K., Grešáková L., Kišidayová S., Babják M., Dolinská M.U., Dvorožňáková E., Königová A. (2019). Natural chemotherapeutic alternatives for controlling haemonchosis in sheep. BMC Vet. Res..

[34] Oliveira Santos F., Ponce Morais Cerqueira A., Branco A., Batatinha M.J.M., Borges Botura M. (2019). Anthelmintic activity of plants against gastrointestinal nematodes of goats: A review. Parasitology.

[35] Hoste H., Torres-Acosta J.F.J., Sandoval-Castro C.A., Mueller-Harvey I., Sotiraki S., Louvandini H., Thamsborg S.M., Terrill T.H. (2015). Tannin containing legumes as a model for nutraceuticals against digestive parasites in livestock. Vet. Parasitol..

[36] Trejo I. (1999). Características del medio físico de la selva baja caducifolia de México. Invest. Geogr..

[37] Rzedowski J. (1991). El endemismo en la flora fanerogámica mexicana: Una apreciación analítica preliminar. Acta Bot. Mex..

[38] Leirana-Alcocer J.L., Hernández-Betancourt S., Salinas-Peba L., Guerrero-González L. (2009). Cambios en la estructura y composición de la vegetación relacionados con los años de abandono de tierras agropecuarias en la selva baja caducifolia espinosa de la reserva de Dzilam, Yucatán. Polibotánica.

[39] Trejo I., Ceballos G., Martínez L., García A., Espinoza E., Creel J.B., Dirzo R. (2010). Las selvas secas del Pacífico Mexicano. Diversidad, Amenazas y Áreas Prioritarias Para la Conservación de Las Selvas Secas Del Pacífico de México.

[40] Ancona J.J., Ruenes-Morales R., Huchim-Herrera J., Montañez-Escalante P.I., González-Iturbe J.A. (2019). Woody species structure, diversity and floristic affinities in seasonally dry forest in the Uxmal archeological zone. Trop. Subtrop. Agroecosyst..

[41] Gutiérrez C., Zamora P. (2012). Especies leñosas de la selva baja caducifolia de Xmatkuil, Yucatán, México. Foresta Veracruzana.

[42] Flores S., Durán R., Ortiz J., Durán R., Méndez M. (2010). Comunidades vegetales terrestres. Biodiversidad y Desarrollo Humano en Yucatán.

[43] Ortíz-Ocampo G.I., Sandoval-Castro C.A., González-Pech P.G., Mancilla-Montelongo G., Ventura-Cordero J., Castañeda-Ramírez G.S., Tun-Garrido J., Torres-Acosta J.F.J. Seasonal variation in the bromatological composition and polyphenol content of the leaves of Gymnopodium floribundum Rolfe from a tropical deciduous forest.

[44] Arellano R.J.S., Flores J.S., Tun-Garrido J. (2003). Nomenclatura, Forma de Vida, Uso, Manejo y Distribución de las Especies Vegetales de la Península de Yucatán. Fascículo No. 20. Etnoflora Yucatanense.

[45] Ayala-Burgos A., Cetina-Góngora R., Capetillo-Leal C.M., Zapata-Campos C., Sandoval-Castro C.A. (2006). Composición Química-Nutricional de Árboles Forrajeros. Compilación de Análisis de Laboratorio de Nutrición de la Facultad de Medicina Veterinaria y Zootecnia de la Universidad Autónoma de Yucatán.

[46] González-Pech P., Ventura-Cordero J., Ortiz-Ocampo G., Jaimez-Rodríguez P., Tun J., Sandoval-Castro C., Torres-Acosta F. (2017). Plantas Consumidas por Ovinos y Caprinos en la Selva Baja Caducifolia de Yucatán. Guía ilustrada.

[47] Ortiz-Domínguez G.A., Ventura-Cordero J., González-Pech P.G., Torres-Acosta J.F.J., Capetillo-Leal C.M., Sandoval-Castro C.A. (2017). Nutritional value and in vitro digestibility of legume pods from seven tres species present in the tropical deciduous forest. Trop. Subtrop. Agroecosyst..

[48] Tainton N.M., Tainton N.M. (1999). The ecology of the main grazing lands of South Africa: The savanna biome. Veld Management in South Africa.

[49] Retama-Flores C., Torres-Acosta J.F.J., Sandoval-Castro C.A., Aguilar-Caballero A.J., Cámara-Sarmiento R., Canul-Ku H.L. (2012). Maize supplementation of Pelibuey sheep in a silvopastoral system: Fodder selection, nutrient intake and resilience against gastrointestinal nematodes. Animal.

[50] Gárate-Gallardo L., Torres-Acosta J.F.J., Aguilar-Caballero A.J., Sandoval-Castro C.A., Cámara-Sarmiento R., Canul-Ku H.L. (2015). Comparing different maize supplementation strategies to improve resilience and resistance against gastrointestinal nematode infections in browsing goats. Parasite.

[51] Pichersky E., Lewinsohn E. (2011). Convergent evolution in plant specialized metabolism. Annu. Rev. Plant. Biol..

[52] Dixon R.A., Strack D. (2003). Phytochemistry meets genome analysis, and beyond. Phytochemistry.

[53] Neilson E.H., Goodger J.Q.D., Woodrow I.E., Møller B.L. (2003). Plant chemical defense: At what cost?. Trends Plant Sci..

[54] Hackmann T.J., Spain J.N. (2010). *Invited review*: Ruminant ecology and evolution: Perspectives useful to ruminant livestock research and production. J. Dairy Sci..

[55] Saarinen J., Gordon I.J., Prins H.H.T. (2019). The Palaeontology of Browsing and Grazing. The Ecology of Browsing and Grazing II.

[56] Mlambo V., Marume U., Gajana C.S. (2015). Utility of the browser’s behavioural and physiological strategies in coping with dietary tannins: Are exogenous tannin-inactivating treatments necessary?. S. Afr. J. Anim. Sci..

[57] Villalba J.J., Costes-Thiré M., Ginane C. (2017). Phytochemicals in animal health: Diet selection and trade-offs between costs and benefits. Proc. Nutr. Soc. USA.

[58] Starkey L.A., Pugh D.G., Pugh D.G., Baird A.N., Edmonson M.A., Passler T. (2020). Internal Parasites of Sheep, Goats and Cervids. Sheep, Goat and Cervid Medicine.

[59] Zajac A.M., Garza J. (2020). Biology, Epidemiology, and Control of Gastrointestinal Nematodes of Small Ruminants. Vet. Clin. N. Am. Food Anim. Pr..

[60] Torres-Acosta J.F.J., Jacobs D.E., Aguilar-Caballero A.J., Sandoval-Castro C.A., May-Martínez M., Cob-Galera L.A. (2004). The effect of supplementary feeding on the resilience and resistance of browsing Criollo kids against natural gastrointestinal nematode infections during the rainy season in tropical México. Vet. Parasitol..

[61] Torres-Acosta J.F.J., Hoste H. (2008). Alternative or improved methods to limit gastro-intestinal parasitism in grazing sheep and goats. Small Rum. Res..

[62] Mavrot F., Hertzberg H., Torgerson P. (2015). Effect of gastro-intestinal nematode infection on sheep performance: A systematic review and meta-analysis. Parasit. Vectors..

[63] O’Connor L.J., Walkden-Brown S.W., Kahn L. (2006). Ecology of the free-living stages of major trichostrongylid parasites of sheep. Vet. Parasitol..

[64] Van Dijk J., de Louw M.D.E., Kalis L.P.A., Morgan E.R. (2009). Ultraviolet light increases mortality of nematode larvae and can explain patterns of larval availability at pasture. Int. J. Parasitol..

[65] Hutchings M., Milner J., Gordon I., Kyriazakis I., Jackson F. (2002). Grazing decisions of Soay sheep (Ovis aries) on St. Kilda: A consequence of parasite distribution?. Oikos.

[66] Fox N.J., Marion G., Davidson R.S., White P.C.L., Hutchings M.R. (2013). Modelling parasite transmission in a grazing system: The importance of host behavior and immunity. PLoS ONE.

[67] Hutchings M.R., Judge J., Gordon I.J., Athanasiadou S., Kyriazakis I. (2006). Use of trade-off theory to advance the understanding of herbivore-parasite interactions. Mamm. Rev..

[68] Vlassoff A., Ross A.D. (1982). Biology and population dynamics of the free living stages of gastrointestinal nematodes in sheep. Control of Internal Parasites of Sheep.

[69] Tontini J.F., Poli C.H., Bremm C., de Castro J.M., Fajardo N.M., Sarout B.N., Castilhos Z.M. (2015). Distribution of infective gastrointestinal helminth larvae in tropical erect grass under different pasture type for lambs. Trop. Anim. Health Product..

[70] Tontini J.F., Poli C.H., Hampel V.S., Fajardo N.M., Martins A.A., Minho A.P., Muir J.P. (2019). Dispersal and concentration of sheep gastrointestinal nematode larvae on tropical pastures. Small Rum. Res..

[71] Jaimez-Rodríguez P.R., González-Pech P.G., Ventura-Cordero J., Brito D.R.B., Costa-Junior L.M., Sandoval-Castro C.A., Torres-Acosta J.F.J. (2019). The worm burden of tracer kids and lambs browsing heterogeneous vegetation is influenced by strata harvested and not total dry matter intake or plant life form. Trop. Anim. Health Prod..

[72] Stear M.J., Singleton D., Matthews L. (2011). An evolutionary perspective on gastrointestinal nematodes of sheep. J. Helminthol..

[73] Poulin R. (2007). Are there general laws in parasite ecology?. Parasitology.

[74] Morrill A., Dargent F., Forbes M.R. (2017). Explaining parasite aggregation: More than one parasite species at a time. Int. J. Parasitol..

[75] Torres-Acosta J.F.J., González-Pech P.G., Chan-Pérez J.I., Sandoval-Castro C.A., Estrada-Reyes Z.M., Mendoza-de-Gives P., Ortega-Pierres M.A., Morales-Monto J. (2014). Experiencias en el control alternativo de nematodos gastrointestinales de pequeños rumiantes domésticos en México. Avances en el Estudio de Helmintos Parásitos.

[76] Torres-Acosta J.F.J., Hoste H., Sandoval-Castro C.A., Torres-Fajardo R.A., Ventura-Cordero J., González-Pech P.G., Mancilla-Montelongo M.G., Ojeda-Robertos N.F., Martínez-Ortiz-de-Montellano C. (2019). The art of war against gastrointestinal nematodes in sheep and goat herds of the tropics. Revista Acadêmica Ciência Animal.

[77] Coop R.L., Holmes P.H. (1996). Nutrition and parasite interaction. Int. J. Parasitol..

[78] Bishop S.C. (2012). A consideration of resistance and tolerance for ruminant nematode infections. Front. Genet..

[79] Ríos G., Riley J. (1985). Preliminary studies on the utilization of the natural vegetation in the henequen zone of Yucatán for the production of goats. I. Selection and nutritive value of native plants. Trop. Anim. Health Prod..

[80] González-Pech P.G., Torres-Acosta J.F.J., Sandoval-Castro C.A., Tun-Garrido J. (2015). Feeding behaviour of sheep and goats in a deciduous tropical forest during the dry season: The same menu consumed differently. Small Rumin. Res..

[81] Ventura-Cordero J., González-Pech P.G., Torres-Acosta J.F.J., Sandoval-Castro C.A., Tun-Garrido J. (2019). Sheep and goat browsing a tropical deciduous forest during the rainy season: Why does similar plant species consumption result in different nutrient intake?. Anim. Prod. Sci..

[82] Maddi V.S., Aragade P.D., Digge V.G., Nitalikar M.N. (2007). Importance of Nutraceuticals in health management. Pharmacol. Rev..

[83] Bhattacharya A., Roy D. (2015). Nutraceuticals in Livestock & Poultry.

[84] Patel K., Katole S. (2018). Nutraceuticals and Ruminants nutrition—A review. Livest. Res. Int..

[85] Gupta R.C., Srivastava A., Lall R. (2019). Nutraceuticals in Veterinary Medicine.

[86] Ballou M.A., Davis E.M., Kasl B.A. (2019). Nutraceuticals. An alternative strategy for the use of antimicrobials. Vet. Clin. Food Anim..

[87] Dzanis D.A. (1999). Nutraceuticals in veterinary medicine. Aus. Vet. J..

[88] Alamgir A.N.M., Alamgir A.N.M. (2017). Classification of Drugs, Nutraceuticals, Functional food, and Cosmeceuticals; Proteins, Peptides, and Enzymes as drugs. Therapeutic Use of Medicinal Plants and their Extracts.

[89] Boothe D.M. (1997). Nutraceuticals in veterinary medicine. Part 1. Definitions and regulations. Compend. Contin. Educ. Vet..

[90] McCorkle C.M. (1986). An introduction to ethnoveterinary research and development. J. Ethnobiol..

[91] Suroowan S., Mahomoodally F., McGaw L., Abdalla M.A. (2020). Alternative Antimicrobials: Medicinal Plants and their Influences on Animal Infectious Diseases. Ethnoveterinary Medicine: Present and Future Concepts.

[92] Andlauer W., Fürst P. (2002). Nutraceuticals: A piece of history, present status and outlook. Food Res..

[93] Agreil C., Meuret M. (2004). An improve method for quantifying intake rate and ingestive behavior of ruminants in diverse and variable habitats using direct observation. Small Rum. Res..

[94] Bonnet O., Meuret M., Tischler M., Cezimbra I., Azambuja J., Carvalho C. (2015). Continuous bite monitoring: A method to assess the foraging dynamics of herbivores in natural grazing conditions. Anim. Prod. Sci..

[95] Illius A.W., Hodgson J., Illius A.W., Hodgson J. (1996). Progress in understanding the ecology and management of grazing systems. The Ecology and Management of Grazing Systems.

[96] Agreil C., Meuret M., Fritz H., Bels V. (2006). Adjustment of feeding choices and intake by a ruminant foraging in varied and variable environments: New insights from continuous bite monitoring. Feeding of Domestic Vertebrates: From Structure to Behavior.

[97] Reppert J.N. (1960). Forage preference and grazing habits of cattle at the Eastern Colorado Range Station. J. Range Manag..

[98] Villalba J.J., Provenza F.P. (2000). Postingestive feedback from starch influences the ingestive behavior of sheep consuming wheat straw. Appl. Anim. Behav. Sci..

[99] Damiram D., DelCurto T., Findholt S.L., Johnson B.K., Vavra M. (2013). Comparison of bite-count and rumen evacuation techniques to estimate cattle diet quality. Rangel. Ecol. Manag..

[100] Halls L.K. (1954). The approximation of cattle diet through herbage sampling. J. Range Manag..

[101] De Vries M. (1995). Estimating forage intake and quality in grazing cattle: A reconsideration of the hand-plucking method. J. Range Manag..

[102] Franco-Guerra F.J., Gómez A.G., Villareal O.A., Camacho J.C., Hernández J.E., Rodríguez E.L., Marcito A. (2014). Tasa de bocados en la vegetación nativa por cabras en pastoreo trashumante en agostaderos montañosos del nudo mixteco, México. Trop. Subtrop. Agroecosyst..

[103] Franco-Guerra F.J., Sánchez M., Camacho J.C., Hernández J.E., Villareal O.A., Rodríguez E.L., Marcito O. (2014). Consumo de especies arbóreas, arbustivas y sus frutos y herbáceas por cabras en pastoreo trashumante en la mixteca oaxaqueña, México. Trop. Subtrop. Agroecosyst..

[104] Albores-Moreno S., Alayón-Gamboa J.A., Morón-Ríos A., Ortiz-Colin P.N., Ventura-Cordero J., González-Pech P.G., Mendoza-Arroyo G.E., Ku-Vera J.C., Jiménez-Ferrer G., Piñeiro-Vázquez A.T. (2020). Influence of the composition and diversity of tree fodder grazed on the selection and voluntary intake by cattle in a tropical forest. Agroforest. Syst..

[105] Manousidis T., Kyriazopoulos A.P., Parissi Z.M., Abraham E.M., Korakis G., Abas Z. (2016). Grazing behavior, forage selection and diet composition of goats in a mediterranean Woody rangeland. Small Rum. Res..

[106] Chebli Y., El Otmani S., Chentouf M., Hornick J.L., Bindelle J., Cabaraux J.F. (2020). Foraging behavior of goats browsing in Southern Mediterranean Forest Rangeland. Animals.

[107] Githiori J.B., Höglund J., Waller P.J. (2005). Ethnoveterinary plant preparations as livestock dewormers: Practices, popular beliefs, pitfalls and prospect for the future. Anim. Health Res. Rev..

[108] Attindéhou S., Houngnimassoun M.A., Salifou S., Biaout C.F. (2012). Inventory of herbal remedies used to control small ruminant’s parasites in Southern Benin. Int. Multidiscip. Res. J..

[109] Vogl C.R., Vogl-Lukasser B., Walkenhorst M. (2016). Local knowledge held by farmers in Eastern Tyrol (Austria) about the use of plants to maintain and improve animal health and welfare. J. Ethnobiol. Ethnomed..

[110] Gradé J.T., Tabuti J.R.S., van Damme P. (2009). Four footed pharmacists: Indication of self-medicating livestock in Karamoja, Uganda. Econ. Bot..

[111] Kahiya C., Mukaratirwa S., Thamsborg S.M. (2003). Effects of *Acacia nilotica* and *Acacia karoo* diets on *Haemonchus contortus* infection in goats. Vet. Parasitol..

[112] Debela E., Tolera A., Olav L., Salte R. (2012). Condensed tannins from *Sebasnia sesban* and *Desmodium intortum* as a means of *Haemonchus contortus* control in goats. Trop. Anim. Health Prod..

[113] Landau S., Azaizeh H., Muklada H., Glasser T., Ungar E.D., Baram H., Abbas N., Markovics A. (2010). Anthelmintic activity of *Pistacia lentiscus* foliage in two Middle Eastern breeds of goats differing in their propensity to consume tannin-rich browse. Vet. Parasitol..

[114] Raju J., Sahoo B., Chandrakar A., Sankar M., Garg A.K., Sharma A.K., Pandey A.B. (2015). Effect of feeding oak leaves (*Quercus semecarpifolia* vs *Quercus leucotricophora*) on nutrient utilization, growth performance and gastrointestinal nematodes of goats in temperate sub Himalayas. Small Rum. Res..

[115] Paolini V., De La Farge F., Prevot F., Dorchies P.H., Hoste H. (2005). Effects of the repeated distribution of sainfoin hay on the resistance and the resilience of goats naturally infected with gastrointestinal nematodes. Vet. Parasitol..

[116] Moreno-Gonzalo J., Ferre I., Celaya R., Frutos P., Ferreira L.M.M., Hervás G., García U., Ortega-Mora L.M., Osoro K. (2012). Potential use of heather to control gastrointestinal nematodes in goats. Small Rum. Res..

[117] Moreno-Gonzalo J., Osoro K., García U., Frutos P., Celaya R., Ferreira L.M.M., Ortega-Mora L., Ferre I. (2014). Anthelmintic effect of heather in goats experimentally infected with *Trichostrongylus colubriformis*. Parasitol. Res..

[118] Moreno F.C., Gordon I.J., Knox M.R., Summer P.M., Skerrat L.F., Benvenutti M.A., Saumell C.A. (2012). Anthelmintic efficacy of five tropical native Australian plants against *Haemonchus contortus* and *Trichostrongylus colubriformis* in experimentally infected goats (*Capra hircus*). Vet. Parasitol..

[119] Shaik S.A., Terrill T.H., Miller J.E., Kouakou B., Kannan G., Kaplan R.M., Burke J.M., Mosjidis J.A. (2006). Sericea lespedeza hay as a natural deworming agent against gastrointestinal nematode infection in goats. Vet. Parasitol..

[120] Gujja S., Terrill T.H., Mosjidis J.A., Miller J.E., Mechineni A., Kommuru D.S., Shaik S.A., Lambert B.D., Cherry N.M., Burke J.M. (2013). Effect of supplemental sericea lespedeza leaf meal pellets on gastrointestinal nematode infection in grazing goats. Vet. Parasitol..

[121] Brito D.R.B., Costa-Junior L.M., García J.L., Torres-Acosta J.F.J., Louvandini H., Cutrim-Júnior J.A.A., Araújo J.F.M., Soares E.D.S. (2018). Supplementation with dry Mimosa caesalpiniifolia leaves can reduce the *Haemonchus contortus* worm burden of goats. Vet. Parasitol..

[122] Lima P.M.T., Crouzolon P., Sanchez T.P., Zabré G., Kabore A., Niderkorn V., Hoste H., Amarante A.F.T., Costa-Junior L.M., Abdalla A.L. (2019). Effects of Acacia mearnsii supplementation on nutrition, parasitological, blood parameters and methane emissions in Santa Inês sheep infected with Trichostrongylus colubriformis and Haemonchus contortus. Exp. Parasitol..

[123] Rojas D., Cubides J., Montenegro A., Martínez C., Ríos de Álvarez L. *In vitro* anthelmintic effect of four extracts obtained from *Caesalpinia coriaria* foliage against an *Haemonchus contortus* isolate. 2. Larvae exsheathment inhibition test. *Poster Presentation*. Proceedings of the 27th Meeting of the World Association for the Advancement of Veterinary Parasitology (WAAVP).

[124] Romero N., Areche C., Cubides-Cardenas J., Escobar N., García-Beltrán O., Simirgiotis M.J., Céspedes A. (2020). In vitro anthelmintic evaluation of *Gliricidia sepium, Leucaena leucocephala*, and *Phitecellobium dulce*: Fingerprint analysis extracts by UHPLC-Orbitrap mass spectrometry. Molecules.

[125] González-Cortázar M., Zamilpa A., López-Arellano M.E., Aguilar-Marcelino L., Reyes-Guerrero D.E., Olazarán-Jenkins S., Ramírez-Vargas G., Olmedo-Juárez A., Mendoza-de-Gives P. (2017). Lysiloma acapulcensis leaves contain anthelmintic metabolites that reduce the gastrointestinal nematode egg population in sheep faeces. Com. Clin. Path..

[126] Higuera-Piedrahita R.I., López-Arellano M.E., López-Arellano R., Cuenca-Verde C., Cuéllar-Ordaz J.A. (2020). Artemisia cina 30 CH como tratamiento homeopático contra el *Haemonchus contortus*. Rev. Mex. Cienc. Pecuarias..

[127] Ortiz-Ocampo G.I., Torres-Acosta J.F.J., Sandoval-Castro C.A., Hoste H., Capetillo-Leal C.M., González-Pech P.G., Santos-Ricalde R.H. (2016). *In vitro* and *in vivo* anthelmintic effect of *Coffea arabica* residues against an *Haemonchus contortus* isolate with low susceptibility to tannins. Trop. Subtrop. Agroecosyst..

[128] Mancilla-Montelongo G., Castañeda-Ramírez G.S., Gaudin E., Canul-Velázquez M.L., Chan-Pérez J.I., De La Cruz-Cortázar A., Mathieu C., Fourquaux I., Sandoval-Castro C.A., Hoste H. *In Vitro* evaluation of the nutraceutical potential of *Theobroma cacao* pod husk and leaf extracts for ruminants.

[129] Ventura-Cordero J., González-Pech P.G., Sandoval-Castro C.A., Torres-Acosta J.F.J., Tun-Garrido J. (2018). Feed resource selection by Criollo goats browsing a tropical deciduous forest. Anim. Prod. Sci..

[130] Torres-Fajardo R.A., Navarro-Alberto J.A., Ventura-Cordero J., González-Pech P.G., Sandoval-Castro C.A., Chan-Pérez J.I., Torres-Acosta J.F.J. (2019). Intake and selection of goats grazing heterogeneous vegetation: Effect of gastrointestinal nematodes and condensed tannins. Rangel. Ecol. Manag..

[131] Karfs R.A., Abbott B.N., Scarth P.F., Wallace J.F. (2009). Land condition monitoring information for reef catchments: A new era. Rangel. J..

[132] Sharma O.P., Sharma S., Pattabhi V., Mahato S.B., Sharma P.D. (2007). A review of the hepatotoxic plant Lantana camara. Crit. Rev. Toxicol..

[133] Ávila-Cervantes R. (2020). Caracterización Nutricional y Fitoquímica In Vitro de 10 Plantas de Bajo o Nulo Consumo de la Selva Baja Caducifolia. Master’s Thesis.

[134] Gusha J., Masocha M., Muchaya M., Ncube S. (2016). Chemical analysis of the potential contribution of Lantana camara to the nutrition of browsing livestock. Trop. Subtrop. Agr..

[135] Hussein R.A., El-Anssary A. (2019). Plants secondary metabolites: The key drivers of the pharmacological actions of medicinal plants. Herbal Medicine.

[136] Barry T.N., Nolan J.V., Leng R.A., Demeyer D.I. (1989). Condensed tannins their role in ruminant protein and carbohydrate digestion and possible effects upon the rumen ecosystem. The Roles of Protozoa and Fungi in Ruminant Digestion.

[137] Alonso-Díaz M.A., Torres-Acosta J.F.J., Sandoval-Castro C.A., Capetillo-Leal C.M. (2010). Polyphenolic compounds of nutraceutical trees and the variability of their biological activity measured by two methods. Trop. Subtrop. Agroecosyst..

[138] Hove L., Ndlovu L.R., Sibanda S. (2003). The effects of drying temperature on chemical composition and nutritive value of some tropical fodder shrubs. Agrofor. Syst..

[139] Ferreira E.C., Nogueira A.R., Souza G.B., Batista L.A. (2004). Effect of drying method and length of storage on tannin and total phenol concentrations in *Pigeon pea* seeds. Food Chem..

[140] Zeller W.E. (2019). Activity, purification, and analysis of condensed tannins: Current state of affairs and future endeavors. Crop Sci..

[141] Kaplan R.M., Vidyashankar A.N. (2012). An inconvenient truth: Global worming and anthelmintic resistance. Vet. Parasitol..

[142] Torres-Acosta J.F.J., Mendoza-de-Gives P., Aguilar-Caballero A.J., Cuéllar-Ordaz J.A. (2012). Anthelmintic resistance in sheep farms: Update of the situation in the American continent. Vet. Parasitol..

[143] Paraud C., Chartier C., Simões J., Gutiérrez C. (2017). Facing Anthelmintic Resistance in Goats. Sustainable Goat Production in Adverse Environments: Volume I, Welfare, Health and Breeding.

[144] Scott I., Pomroy W.E., Kenyon P.R., Smith G., Adlington B., Moss A. (2013). Lack of efficacy of monepantel against *Teladorsagia circumcinta* and *Trichostrongylus colubriformis*. Vet. Parasitol..

[145] Van de Brom R., Moll L., Kappert C., Vellema P. (2015). *Haemonchus contortus* resistance to monepantel in sheep. Vet. Parasitol..

[146] Wood I.B., Amaral N.K., Bairden K., Duncan J.L., Kassai T., Malone J.B., Pankavich J.A., Reinecke R.K., Slocombe O., Taylor S.M. (1995). World Association for the Advancement of Veterinary Parasitology (W.A.A.V.P.) second edition of guidelines for evaluating the efficacy of anthelmintics in ruminants (bovine, ovine, caprine). Vet. Parasitol.

[147] Bauhaud D., Martínez-Ortiz-De-Montellano C., Chauveau S., Prevot F., Torres-Acosta J.F.J., Fouraste I., Hoste H. (2006). Effects of four tanniniferous plant extracts on the in vitro exsheatment of third-stage larvae of parasitic nematodes. Parasitology.

[148] Jackson F., Hoste H., Vercoe P.E., Makkar H.P.S., Schlink A.C. (2010). In vitro methods for the primary screening of plant products for direct activity against ruminant gastrointestinal nematodes. In Vitro Screening of Plant Resources for Extra Nutritional Attributes in Ruminants: Nuclear and Related Methodologies.

[149] Higuera-Piedrahita R.I., López-Arellano M.E., López-Arellano R., Cuenca-Verde C., Cuéllar-Ordaz J.A. (2016). Effect evaluation of artemisins from ethanolic extract of *Artemisia cina* against L3 of *Haemonchus contortus* on a abomasal explants technique. Rev. Cien. Agri..

[150] Quijada J.C., Fryganas C., Ropiak H.M., Ramsay A., Mueller-Harvey I., Hoste H. (2015). Anthelmintic activities against *Haemonchus contortus* or *Trichostrongylus colubriformis* from small ruminants are influenced by structural features of condensed tannins. J. Agric. Food Chem..

[151] Borges D.G.L., Borges F.A. (2016). Plants and their medicinal potential for controlling gastrointestinal nematodes in ruminants. Nematoda.

[152] Villalba J.J., Provenza F.D., Vercoe P.E., Makkar H.P.S., Schlink A.C. (2010). Challenges in Extrapolating In vitro Findings to In Vivo Evaluation of Plant Resources. In Vitro Screening of Plant Resources for Extra Nutritional Attributes in Ruminants: Nuclear and Related Methodologies.

[153] Meier J.S., Kreuzer M., Marquardt S. (2012). Design and methodology of choice feeding experiments with ruminant livestock. Appl. Anim. Behav. Sci..

[154] Ben Salem H., Nefzaoui A., Abdouli H. (1994). Palatability of shrubs and fodder trees measured on sheep and dromedaries: 1. Methodological approach. Anim. Feed. Sci. Technol..

[155] Coop R.L., Kyriazakis I. (1999). Nutrition-parasite interaction. Vet. Parasitol..

[156] Coop R.L., Kyriazakis I.K. (2001). Influence of host nutrition on the development and consequences of nematode parasitism in ruminants. Trends Parasitol..

[157] Hoste H., Torres-Acosta J.F.J., Quijada J., Chan-Pérez I., Dakheel M.M., Kommuru D.S., Mueller-Harvey I., Terrill T.H. (2016). Interactions between nutrition and infections with *Haemonchus contortus* and related gastrointestinal nematodes in small ruminants. Adv. Parasitol..

[158] Mohrand-Fehr P. (2005). Recent development in goat nutrition and application: A review. Small Rumin. Res..

[159] Mancilla-Montelongo G.M., Castañeda-Ramírez G.S., Can-Celis A., Chan-Pérez J.I., Sandoval-Castro C.A., Torres-Acosta J.F.J. (2020). Optimal age of *Trichostrongylus colubriformis* larvae (L_3_) for the in vitro larval exsheathment inhibition test under tropical conditions. Vet. Parasitol..

[160] Perevolotsky A., Landau S., Silanikove N., Provenza F., Sandoval-Castro C.A., Deb Hovell F.V., Torres-Acosta J.F.J., Ayala-Burgos A. (2006). Upgrading tannin-rich forages by supplementing ruminants with polyethylene glycol (PEG). Herbivores: The Assessment of Intake, Digestibility and the Roles of Secondary Compounds.

[161] Bhat T.K., Kannan A., Singh B., Sharma O.P. (2013). Value addition of feed and fodder by alleviating the antinutritional effects of tannins. Agric. Res..

[162] Jackson F., Varady M., Bartley D.J. (2012). Managing anthelmintic resistance in goats—Can we learn lessons from sheep?. Small Rum. Res..

[163] Lespine A., Chartier C., Hoste H., Alvinerie M. (2012). Endectocides in goats: Pharmacology, efficacy and use conditions in the context of anthelmintics resistance. Small Rum. Res..

[164] Rostang A., Devos J., Chartier C.H. (2020). Review of the Eprinomectin effective doses required for dairy goats: Where do we go from here?. Vet. Parasitol..

[165] Codron D., Hofmann R.R., Clauss M., Gordon I.J., Prins H.H.T. (2019). Morphological and Physiological Adaptations for Browsing and Grazing. The Ecology of Browsing and Grazing II.

[166] Gordon I.J., Prins H.H.T., Gordon I.J., Prins H.H.T. (2019). The Ecology of Browsing and Grazing II.

[167] Silanikove N. (2000). The physiological basis of adaptation in goats to harsh environment. Small Rum. Res..

[168] Hofmann R.R. (1989). Evolutionary steps of ecophysiological adaptation and diversification of ruminants: A comparative view of their digestive system. Oecologia.

[169] Pfister J.A., Malecheck J.C. (1986). Dietary selection by goats and sheep in deciduous woodland of northeastern Brazil. J. Range Manag.

[170] Lu C.D. (1988). Grazing behavior and diet selection of goats. Small Rum. Res..

[171] Decandia M., Yiakoulaki M.D., Pinna G., Cabiddu A., Molle G., Cannas A., Pulina G. (2005). Foraging behavior and intake of goats browsing on Mediterranean shrublands. Dairy Goats Feeding and Nutrition.

[172] Dove H., Solaiman S.G. (2010). Ingestive behavior, diet selection, and feed intake. Goat Science and Production.

[173] Goetsch A.L., Gipson T.A., Askar A.R., Puchala R. (2010). Invited review: Feeding behavior of goats. J. Anim. Sci..

[174] Hoste H., Sotiraki S., Landau S.Y., Jackson F., Beveridge I. (2010). Goat-Nematode interactions: Think differently. Trends Parasitol..

[175] Huntley J.F., Patterson M., Mackellar A., Jackson F., Stevenson L.M., Coop R.L. (1995). A comparison of the mast cell and eosinophil responses of sheep and goats to gastrointestinal nematode infections. Res. Vet. Sci..

[176] Silvestre A., Leignel V., Berrag B., Gasnier N., Humbert J.F., Chartier C., Cabaret J. (2002). Sheep and goat nematode resistance to anthelmintics: Pro and cons breeding management factors. Vet. Res..

[177] Amit M., Cohen I., Marcovics A., Muklada H., Glasser T.A., Ungar E.D., Landau S.Y. (2013). Self-medication with tannin rich browse in goats infected with gastrointestinal nematodes. Vet. Parasitol..

[178] Forbes J.M. (2007). Voluntary Intake and Diet Selection in Farm Animals.

[179] Carvalho P. (2013). Harry Stobbs Memorial Lecture: Can grazing behavior support innovations in grassland management?. Trop. Grassl..

[180] Johnson D. (1980). The comparison of usage and availability measurements for evaluating resource preference. Ecology.

[181] Manly B.F.J., McDonald L.L., Thomas D.L., McDonald T.L., Erickson W.P. (2004). Resource Selection by Animals, Statistical Design and Analysis for Field Studies.

[182] Lechowicz M.J. (1982). The sampling characteristics of electivity indexes. Oecologia.

[183] Ventura-Cordero J., González-Pech P.G., Jaimez-Rodríguez P.R., Ortíz-Ocampo G.I., Sandoval-Castro C.A., Torres-Acosta J.F.J. (2017). Gastrointestinal nematode infection does not affect selection of tropical foliage by goats in a cafeteria trial. Trop. Anim. Health Prod..

[184] Ventura-Cordero J., González-Pech P.G., Jaimez-Rodríguez P.R., Ortíz-Ocampo G.I., Sandoval-Castro C.A., Torres-Acosta J.F.J. (2017). Feed resource selection of Criollo goats artificially infected with *Haemonchus contortus*: Nutritional wisdom and prophylactic self-medication. Animal.

[185] Torres-Fajardo R.A., González-Pech P.G., Ventura-Cordero J., Ortíz-Ocampo G.I., Sandoval-Castro C.A., Torres-Acosta J.F.J. (2018). Feed resource selection of Criollo goats is the result of an interaction between plant resources, condensed tannins and *Haemonchus contortus* infection. Appl. Anim. Behav. Sci..

[186] Pisani J.M., Distel R.A., Bontti E.E. (2001). Diet selection by goats on a semi-arid shrubland in central Argentina. Ecol. Austral..

[187] Egea A.V., Allegretti L., Paez Lama S., Grilli D., Sartor C., Fucili M., Guevara J.C., Passera C. (2014). Selective behavior of Creole goats in response to the functional heterogeneity of native forage species in the central Monte desert, Argentina. Small Rum. Res..

[188] Bernhoft A., Bernhoft A. (2010). A brief review on bioactive compounds in plants. Proceedings of the Bioactive compounds in plants—Benefits and risk for man and animals.

[189] Zeineldin M.M., Sabek A.A., Barakat R.A., Elghandour M.M.M.Y., Salem A.Z., Jiménez R.M. (2018). Potential contribution of plants bioactive in ruminant productive performance and their impact on gastrointestinal parasites elimination. Agroforest. Syst..

[190] Eguale T., Tilahun G., Debella A., Feleke A., Makonnen E. (2007). *Haemonchus contortus*: In *Vitro* and In Vivo anthelmintic activity of aqueous and hidro-alcoholic extracts of Hedera helix. Exp. Parasitol..

[191] Marie-Magdeleine C., Udino L., Philibert L., Bocage B., Archimede H. (2010). *In vitro* effects of Cassava (*Manihot esculenta*) leaf extracts on four development stages of *Haemonchus contortus*. Vet. Parasitol..

[192] Klongsiriwet C., Quijada J., Williams A.R., Mueller-Harvey I., Williamson E.M., Hoste H. (2015). Synergistic inhibition of *Haemonchus contortus* exsheathment by flavonoid monomers and condensed tannins. Int. J. Parasitol..

[193] Fomum S.W., Nsahlai I.V. (2017). *In Vitro* nematicidal activity of plant species possessing alkaloids and tannins. Cogent Food Agric..

[194] Spiegler V., Liebau E., Hensel A. (2017). Medicinal plant extracts and plant-derived polyphenols with anthelmintic activity against intestinal nematodes. Nat. Prod. Rep..

[195] Maestrini M., Tava A., Mancini S., Tedesco D., Perrucci S. (2020). *In vitro* anthelmintic activity of saponins from *Medicago spp*. against sheep gastrointestinal nematodes. Molecules.

[196] Zarza-Albarrán M.A., Olmedo-Juárez A., Rojo-Rubio R., Mendoza-de-Gives P., González-Cortázar M., Tapia-Maruri D., Mondragón-Ancelmo J., García-Hernández C., Blé-González E.A., Zamilpa A. (2020). Galloyl flavonoids from *Acacia farnesiana* pods possess potent anthelmintic activity against *Haemonchus contortus* eggs and infective larvae. J. Ethnopharmacol..

[197] Banner R.E., Rogosic J., Burritt E.A., Provenza F.D. (2000). Supplemental barley and charcoal increase intake of sagebrush by lambs. J. Range Manag..

[198] Rogosic J., Moe S.R., Skobic D., Knezovic Z., Rozic I., Rozic I., Zivkovic M., Pavlicevic J. (2009). Effect of supplementation with barley and activated charcoal on intake of biochemically diverse Mediterranean shrubs. Small Rum. Res..

